# *Foxg1* bimodally tunes *L1*-mRNA and -DNA dynamics in the developing murine neocortex

**DOI:** 10.1242/dev.202292

**Published:** 2024-05-23

**Authors:** Gabriele Liuzzi, Osvaldo Artimagnella, Simone Frisari, Antonello Mallamaci

**Affiliations:** Laboratory of Cerebral Cortex Development, SISSA, Trieste 34136, Italy

**Keywords:** Foxg1, L1 retrotransposon, Transcription, Retro-transcription, Cerebral cortex

## Abstract

*Foxg1* masters telencephalic development via a pleiotropic control over its progression. Expressed within the central nervous system (CNS), *L1* retrotransposons are implicated in progression of its histogenesis and tuning of its genomic plasticity. Foxg1 represses gene transcription, and *L1* elements share putative Foxg1-binding motifs, suggesting the former might limit telencephalic expression (and activity) of the latter. We tested such a prediction, *in vivo* as well as in engineered primary neural cultures, using loss- and gain-of-function approaches. We found that *Foxg1*-dependent, transcriptional *L1* repression specifically occurs in neopallial neuronogenic progenitors and post-mitotic neurons, where it is supported by specific changes in the *L1* epigenetic landscape. Unexpectedly, we discovered that Foxg1 physically interacts with *L1*-mRNA and positively regulates neonatal neopallium *L1*-DNA content, antagonizing the retrotranscription-suppressing activity exerted by Mov10 and Ddx39a helicases. To the best of our knowledge, *Foxg1* represents the first CNS patterning gene acting as a bimodal retrotransposon modulator, limiting transcription of *L1* elements and promoting their amplification, within a specific domain of the developing mouse brain.

## Teaser

**Summary:** The brain-patterning gene *Foxg1* is shown to act as a region-specific, bimodal retrotransposon modulator, limiting transcription of *L1* elements and promoting their amplification in the developing rodent CNS.

## INTRODUCTION

Foxg1 encodes an evolutionarily ancient transcription factor that drives the development of the anterior brain (Hanashima et al., 2004). It promotes the activation of subpallial (Martynoga et al., 2005) and neo-paleo-pallial (Muzio and Mallamaci, 2005) morphogenetic programs, regulates pallial stem cells fate choice, promoting neuronogenesis at expenses of gliogenesis (Brancaccio et al., 2010; Falcone et al., 2019; Frisari et al., 2022), and commits neocortical neurons to distinct layer identities (Hanashima et al., 2004; Hou et al., 2019; Miyoshi and Fishell, 2012; Toma et al., 2014). Subsequently, Foxg1 stimulates neuronal morphological maturation (Brancaccio et al., 2010; Chiola et al., 2019; Yu et al., 2019; Zhu et al., 2019), and enhances electrical activity (Tigani et al., 2020; Zhu et al., 2019), being in turn transiently upregulated by the latter (Fimiani et al., 2016; Tigani et al., 2020). Experimental *Foxg1* knockdown *in vivo* reduces social interaction and results in selective impairment of specific learning and memory abilities (Miyoshi et al., 2021; Shen et al., 2006; Yu et al., 2019). In humans, several *FOXG1* copy number variations (CNVs) and structural mutations have been described. They lead to severe neuropathological scenarios, collectively referred to as FOXG1 syndrome, for which no cure is so far available (Brimble et al., 2023; https://www.ncbi.nlm.nih.gov/clinvar/?term=Foxg1[gene]; Florian et al., 2011; Mitter et al., 2018; Papandreou et al., 2016; https://gene.sfari.org/database/human-gene/Foxg1#variants-tab; Vegas et al., 2018). Traditionally recognized as a transcriptional transrepressor (Seoane et al., 2004), Foxg1 has more recently been implicated in the straight control of extra-transcriptional functions, such as post-transcriptional ncRNA processing (Weise et al., 2019), translation (Artimagnella et al., 2022 preprint) and mitochondrial biology (Pancrazi et al., 2015).

Albeit tightly controlled (Faulkner and Billon, 2018; Goodier, 2016), transposable elements, including *L1* retrotransposons, are actively transcribed. Specific ensembles of such elements are activated concomitantly with distinct, early histogenetic routines (He et al., 2021), and, in some cases, their transcription is required for the progression of these routines (Jachowicz et al., 2017; Macfarlan et al., 2012; Percharde et al., 2018). Moreover, a subset of full-length *L1* retrotransposons is able to undergo somatic retrotransposition. There are only ∼145 retrotransposition-competent L1 elements in humans but ∼3000 in mice, including 900, 400 and 1800 elements belonging to A, Gf and Tf families, respectively (An, 2012; Floreani et al., 2022). Products of their somatic retrotransposition generally lack family-specific 5′UTRs (Singer et al., 2010), while retaining shared orf2 and 3′UTR regions. Clonal analysis has robustly demonstrated that somatic retrotransposition takes place within the developing embryo at different times and in variety of cell types, with special emphasis on the developing CNS (Bodea et al., 2018; Evrony et al., 2015; He et al., 2021; Richardson et al., 2014; Zhu et al., 2021). The magnitude of L1 neo-retrotransposition in the human CNS has been hotly debated, and human neocortical/ hippocampal neurons have been reported to harbor somatic L1 insertions at a frequencies of between 0.2 and 80 events per neural cell (Baillie et al., 2011; Coufal et al., 2009; Evrony et al., 2012, 2016; Upton et al., 2015). An increase in *L1*-DNA content (close to +30%) has also been reported in mice, in both neocortical and hippocampal neurons, between embryonic day 15.5 (E15.5) and postnatal day 14 (P14) (Fontana et al., 2021).

A substantial fraction of Foxg1 protein is stably bound to chromatin (De Filippis et al., 2012), suggesting it might be implicated in long-term gene repression. Next, motif enrichment analysis (MEA) of the *L1* consensus sequence using Jaspar software (Mathelier et al., 2016) revealed a high-score, putative FOXG1-binding site (RTAAACAW) within the *L1*-*orf2* coding sequence (G.L., O.A., S.F. and A.M., unpublished). Based on this information, we hypothesized that Foxg1 may be implicated in regulation of *L1* transcription. We tested this hypothesis within the murine embryonic neocortex. We showed that Foxg1-dependent *L1* repression mainly occurs in neuronogenic progenitors and post-mitotic neurons, accompanied by specific changes in the epigenetic landscape. Unexpectedly, we also found that Foxg1 positively influences neopallial *L1*-DNA content, counteracting the retrotranscription-suppressing activity exerted by Mov10 and Ddx39a helicases.

## RESULTS

### *In vivo Foxg1* downregulation of *L1*-mRNA

**Fig. 1.**
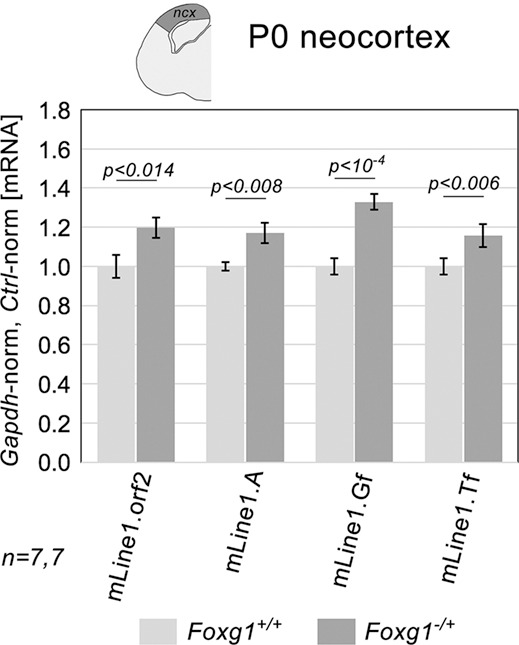
**L1 transcript levels in the neocortex of *Foxg1^−/+^* and control mouse neonates.** RT-PCR quantitation of pan-*L1* diagnostic amplicon orf2 and family-specific amplicons A, Gf and Tf. Data are double normalized against *Gapdh* and wild-type controls. Error bars indicate s.e.m. Statistical significance of results was evaluated by a one-tailed, unpaired *t*-test. *n* is the number of biological replicates, i.e. neocortices taken from distinct pups.

To investigate Foxg1 involvement in regulation of *L1* transcription, we compared *L1*-mRNA levels in the neocortex of P0 *Foxg1^−/+^* mice (Hébert and McConnell, 2000) (Fig. S2A) and wild-type controls. As expected, we observed a substantial upregulation of *L1* expression in these mutants. It was detected by the diagnostic ‘*L1*.orf2’ amplicon, common to all *L1* families, as well by the ‘*L1*.5′UTR.A’, ‘*L1*.5′UTR.Gf’ and ‘*L1*.5′UTR.Tf’ amplicons (hereafter referred to also as *L1.A*, *L1.Gf* and *L1.Tf*), which are specific to their respective transposition-competent families (Fig. S1; Table S1; Sookdeo et al., 2013; Storer et al., 2021). The magnitudes of the upregulation were +19.7±1.6% (*P*<0.014, *n*=7, +17.2±0.3% (*P*<0.008, *n*=7), +33.0±0.1% (*P*<10^−4^, *n*=7) and +15.8±0.5% (*P*<0.006, *n*=7), as for *L1.orf2*, *L1.A*, *L1.Gf* and *L1.Tf*, respectively (Fig. 1).

### *In vitro* modeling of *L1-*mRNA progression in murine developing neocortex

**Fig. 2.**
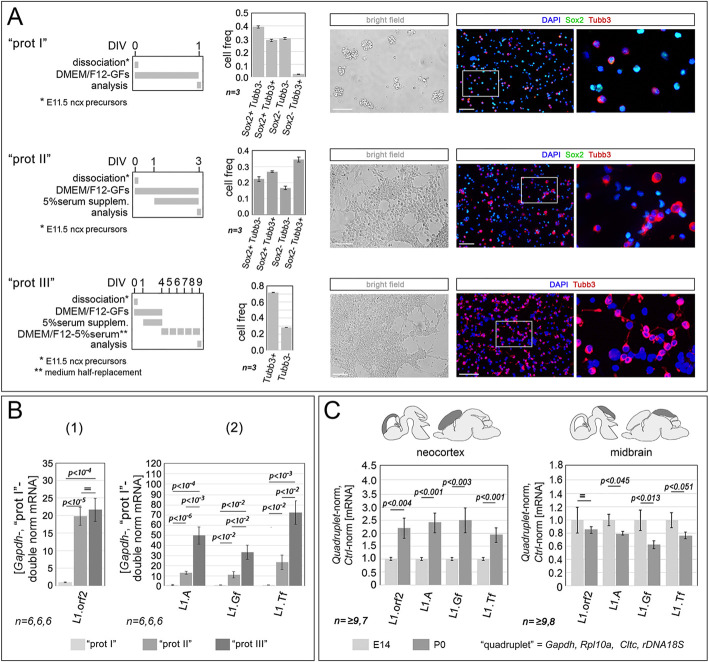
***In vitro* modeling of *L1*-mRNA progression in murine developing neocortex.** (A) Schematics of the three protocols (type I, II and III) employed to generate primary cultures, which model early, mid and late phases of neuronogenesis, respectively, and include neural cells terminally exposed to growth factors (GFs), GFs and serum, and serum, respectively. Graphs show the prevalence of distinctive cell types forming these cultures: type I cultures are enriched with neural stem cells (NSCs, Sox2^+^Tubb3^−^) and neuronogenic progenitors (NPs, Sox2^−^Tubb3^−^); type II cultures include comparable fractions of NSCs, NPs and neurons (Ns, Tubb3^+^); and type III cultures are highly enriched with Ns. Low-Sox2-expressing cells (<2×background) are classified as Sox2^−^, whereas all cells classified as Sox2^+^ expressed the protein at much higher levels (>5×background). Images show examples of primary data; these include bright-field pictures of living cultures, taken immediately before their terminal analysis, and dark-field images of aSox2/aTubb3 immunofluorescence, performed upon culture dissociation and fixation. (B) RT-PCR quantitation of pan-*L1* diagnostic amplicon orf2 (1), and family-specific amplicons, A, Gf and Tf (2), in neural cultures set according to protocols I, II and III. Data are double normalized against *Gapdh* and protocol I values. (C) RT-PCR quantitation of the same diagnostic amplicons in neocortex and mesencephalic tectum at embryonic day 14.5 (E14.5) and birth (P0). Data are double normalized against the geometric mean of *Gapdh*, *Rpl10a*, *Cltc* and *rDNA18S* (quadruplet), and E14.5 values. Error bars indicate s.e.m. Statistical significance of results was evaluated using a one-tailed, unpaired *t*-test. *n* is the number of biological replicates, i.e. independently cultured cell aliquots, originating from pooled, wild-type E11.5 neocortical primordia (B) or distinct embryos/pups (C).

To ease the dissection of Foxg1 control over *L1* expression, we established three protocols, termed type I, type II and type III, for the generation of primary neural cultures representing early, mid and late phases of pallial neuronogenesis, respectively (Fig. 2A). Characterized by progressively longer durations, such protocols differed for terminal exposure of neural cells to ‘pure pro-proliferative’, ‘mixed pro-proliferative/pro-differentiative’ and ‘pure pro-differentiative’ media, respectively. Neural cells generated by these protocols were classified based on their Sox2/Tubb3 expression profiles (Hutton and Pevny, 2011; Menezes and Luskin, 1994). Type I cultures predominantly comprised Sox2^+^Tubb3^−^ presumptive neural stem cells (NSCs; 39.3±0.9%; *n*=3) and Sox2^−^Tubb3^−^ neuronogenic progenitors (NPs; 30.1±0.7%; *n*=3), with a limited Tubb3^+^ neuronal output (30.6±1.2%; *n*=3). The prevalence of these two precursors decreased (to 22.2±1.4% and 16.6±1.1%, respectively; *n*=3) in type II cultures, which were characterized by more frequent neurons (Ns; 61.2±2.0%; *n*=3). As expected, neuronal prevalence further increased in type III cultures (71.7±1.0%; *n*=3).

Subsequently, we profiled these cultures for *L1*-mRNA expression levels using qRT-PCR (Fig. 2B). A progressive increase in the expression of the pan-*L1* diagnostic amplicon ‘*L1.orf2*’, was observed moving from type I to type II and type III cultures [type I culture-normalized values were: 1.00±0.11 (*n*=6), 19.85±2.59 (*n*=6) and 21.65±3.27 (*n*=6), respectively, with *P*_(type I - vs - type II)_<10^−5^, *P*_(type II -vs - type III)_<0.34]. Similar progressions were also observed in cases of family-specific amplicons, namely *L1.5'UTR.A* [type I culture-normalized values: 1.00±0.39 (*n*=6), 13.01±1.49 (*n*=6) and 49.87±8.57 (*n*=6), respectively, with *P*_(type I - vs - type II)_<10^−5^, *P*_(type II - vs - type III)_<10^−3^], *L1.5′UTR.Gf* [type I culture-normalized values: 1.00±0.10 (*n*=5), 11.16±3.10 (*n*=6) and 33.31±7.14 (*n*=6), respectively, with *P*_(type I - vs - type II)_<10^−2^, *P*_(type II - vs - type III)_<10^−2^] and *L1.5'UTR.Tf* [type I culture-normalized values: 1.00±0.05 (*n*=5), 23.41±7.26 (*n*=6) and 72.48±11.49 (*n*=5), respectively, with *P*_(type I - vs - type II)_<10^−2^, *P*_(type II - vs - type III)_<10^−2^]. This scenario indicates a generalized upregulation of *L1* expression, which is associated with neocortical neuronogenesis progression.

To validate the biological plausibility of these results, we repeated this analysis *in vivo*, by comparing *L1* expression in neocortical tissue taken from E14.5 (mid-neuronogenic) and P0 (post-neuronogenic) mice (Fig. 2C). [Here, to enhance the robustness of the results, we normalized *L1* qRT-PCR values against a specific ‘gene quadruplet’. This included three RNA-pol II-transcribed genes (*Gapdh*, *Rpl10a* and *Cltc*), characterized by comparable expression profiles in apical precursors (APs), basal progenitors (BPs), early neurons (eNS) and late neurons (lNs) (Telley et al., 2016) (Table S3A), as well as by poor sensitivity to *Foxg1* manipulation (Artimagnella and Mallamaci, 2020) (Table S3B). Additionally, the quadruplet also included RNA-pol I*-*transcribed *rDNA-45S*, from which the large majority of cell RNA complement is generated.] As expected, we observed a robust upregulation of *L1*-mRNAs in P0 compared with E14.5 neocortices, as evidenced by the amplicons *L1.orf2* [+122.3±47.7%, *P*<0.004, *n*=10 (P0), *n*=7 (E14.5)], *L1.5*′*UTR.A* [+140.6±44.0%, *P*<0.001, *n*=10 (P0), *n*=7 (E14.5)], *L1.5*′*UTR.Gf* [+149.6±51.4%, *P*<0.003, *n*=9 (P0), *n*=7 (E14.5)] and *L1.5*′*UTR.Tf* [+91.0±28.1%, *P*<0.001, *n*=10 (P0), *n*=7 (E14.5)]. As a specificity control, a similar analysis was performed on the mesencephalic tectum (a CNS district not expressing *Foxg1*), harvested from the same animals. Intriguingly, this revealed an opposite E14.5→P0 dynamic in *L1*-mRNA levels [*L1.5*′*UTR.A*: −20.9±5.1%, *P*<0.045, *n*=8 (P0), *n*=9 (E14.5); *L1.5*′*UTR.Gf*: −36.5±4.7%, *P*<0.013, *n*=8 (P0), *n*=10 (E14.5); *L1.5*′*UTR.Tf*: −24.3±5.3%, *P*<0.051, *n*=8 (P0), *n*=10 (E14.5).

### Modeling *Foxg1* regulation of *L1*-mRNA

**Fig. 3.**
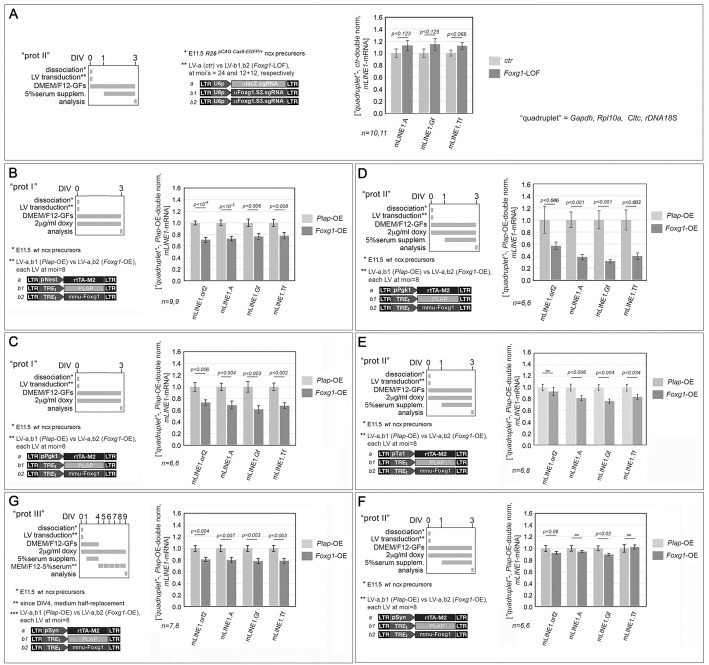
**Impact of *Foxg1* manipulation on *L1*-mRNA levels in progressively more advanced neuronogenic pallial cultures.** (A-G) Outcomes of *Foxg1* downregulation (A, *Foxg1*-LOF) and overexpression (B-G, *Foxg1*-OE) in early- (B,C), mid- (A,D-F) and late- (G) neuronogenic cultures, set according to type I, II and III protocols, respectively. Schematics of the protocols and lentiviral vectors used are on the left. Transgenes driven by pNes, pTα1 and pSyn promoters, which are active in NSCs, NPs/Ns and Ns, respectively, and ubiquitously firing U6p and pPgk1 promoters were used. Graphs show RT-PCR quantitation of pan-*L1* diagnostic amplicon orf2, and family-specific amplicons A, Gf and Tf, in neural cultures set according to the above-mentioned protocols. Data are double normalized against gene quadruplet (*Gapdh*, *Rpl10a*, *Cltc* and *rDNA 18S*) and control values. Error bars indicate s.e.m. Statistical significance of results was evaluated using a one-tailed, unpaired *t*-test. *n* is the number of biological replicates, i.e. independently cultured and engineered cell aliquots, originating from pooled *R26^pCAG-Cas9-EGFP/+^* E11.5 neocortical primordia.

To dissect Foxg1 control over *L1* transcription, we first evaluated *L1*-mRNA levels in type II, mid-neuronogenic cultures, where *Foxg1* had been constitutively knocked down (Table S2 and Fig. S2B) by CRISPR-Cas9 technology and lentiviral transgenesis (Fig. 3A). Consistent with findings in P0 *Foxg1^−/+^* pups (Fig. 1) and upon normalization against the *Gapdh*, *Rpl10a*, *Cltc* and *rDNA18S* quadruplet, neocortical *Foxg1* loss-of-function (LOF) cultures exhibited an increasing trend in *L1*-mRNAs from all three families; however it was not statistically significant (Fig. 3A and Fig. S4A).

Next, we examined *L1*-mRNA response to Foxg1 upregulation. To obtain insights into temporal and intra-neuronogenic lineage progression of Foxg1 modulation of *L1* expression, we run multiple *Foxg1*-overexpression (OE) assays, employing different, early-, mid- and late-neuronogenic, cultures, and driving the *Foxg1* transgene by means of the ubiquitous pPgk1 promoter and the cell type-specific, pNes, pTa1 and pSyn promoters, which are active in NSCs, NPs/Ns and Ns, respectively (Brancaccio et al., 2010; Tigani et al., 2020). As a control, we used the *ALPP* gene, encoding human placental alkaline phosphatase, hereafter referred to as *Plap* (Falcone et al., 2016).

Foxg1-OE in early-neuronogenic (type I) cultures, driven by either pNes or pPgk1, resulted in a similar and generalized downregulation of *L1*-mRNAs. Specifically, in the case of pNes-manipulated cultures, L1.orf2, .A, .Gf and .Tf signals were decreased by 29.1±4.1% (*P*<10^−4^, *n*=9), 27.3±3.8% (*P*<10^−3^, *n*=9), 23.2±5.0% (*P*<0.006, *n*=9), and 22.3±5.5% (*P*<0.008, *n*=9), respectively (Fig. 3B). Similarly, in pPgk1-manipulated cultures,these signals were reduced by 26.8±4.5% (*P*<0.006, *n*=6), 31.0±6.7% (*P*<0.004, *n*=6), 38.5±6.3% (*P*<0.003, *n*=6), and 32.2±5.0% (*P*<0.002, *n*=6), respectively (Fig. 3C).

Conversely, mid-neuronogenic (type II) *Foxg1*-OE cultures showed different results depending on the promoter driving the *Foxg1* transgene. The most pronounced *L1* downregulation was observed in *Foxg1*-OE^pPgk1^ cultures, with *L1*.orf2, .A, .Gf, and .Tf signals decreased by 42.9±6.1% (*P*<0.046, *n*=6), 61.4±4.4% (*P*<0.001, *n*=6), 68.1±2.9% (*P*<0.001, *n*=6) and 60.2±5.4% (*P*<0.003, *n*=6), respectively (Fig. 3D). A milder decline in *L1-*mRNA was observed in *Foxg1*-OE^pTa1^ cultures, where L1.A, .Gf, and .Tf signals decreased by 18.1±4.6% [*P*<0.025, *n*=7 (ctrl), *n*=5 (*Foxg1*-OE^pTa1^)], 23.4±3.5% [*P*<0.004, *n*=7 (ctrl), *n*=5 (*Foxg1*-OE^pTa1^)] and 16.1±4.3% [*P*<0.034, *n*=7 (ctrl), *n*=5 (*Foxg1*-OE^pTa1^)], respectively (Fig. 3E). *L1*-mRNA levels were mostly unaffected in *Foxg1*-OE^pSyn^ type II cultures, except for the *L1*.Gf signal, which was reduced by 10.7±0.8% [*P*<0.024, *n*=6 (ctrl), *n*=5 (*Foxg1*-OE^pSyn^)] (Fig. 3F).

Finally, late-neuronogenic cultures (type III), again over-expressing *Foxg1* under the control of the pSyn promoter however over a longer duration, displayed a generalized *L1*-mRNA downregulation, with L1.orf2, .A, .Gf, and .Tf signals decreased by 19.2±3.8% [*P*<0.004, *n*=7 (ctrl), *n*=8 (*Foxg1*-OE^pSyn^)], 14.3±6.7% [*P*<0.007, *n*=7 (ctrl), *n*=8 (*Foxg1*-OE^pSyn^)], 15.6±7.2% [*P*<0.003, *n*=7 (ctrl), *n*=8 (*Foxg1*-OE^pSyn^)], and 21.3±4.0% [*P*<0.003, *n*=7), respectively (Fig. 3G).

Although pointing to a general trend of *Foxg1*-dependent *L1* downregulation, these results offer valuable insights into temporal and cell-type specific unfolding of this process. In this respect, it is important to distinguish between late neuronogenic cultures manipulated by a *pSyn-driven Foxg1* transgene (Fig. 3G) and other cultures (Fig. 3B-F).

**Fig. 4.**
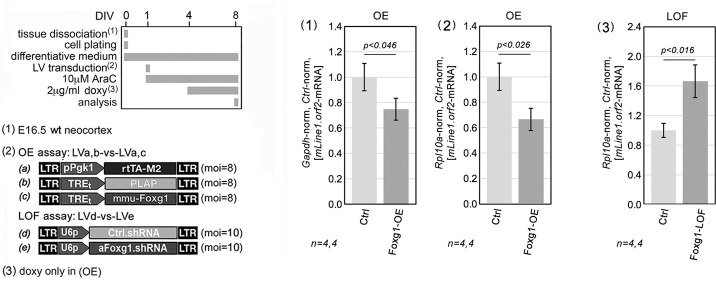
**Impact of constitutive *Foxg1* overexpression on *L1*-mRNA levels in primary neuron-enriched cultures.** Schematics of the protocols and lentiviral vectors employed are on the left. Graphs show neuronal enrichment obtained by early AraC supplementation. Foxg1 was constitutively overexpressed (OE) by a pPgk1-driven transgene or downregulated (LOF) by RNAi. *L1*-mRNA data are double normalized against *Rpl10a* (or *Gapdh*) and control samples. Error bars indicate s.e.m. Statistical evaluation of results was evaluated using a one-tailed, unpaired *t*-test. *n*=number of biological replicates, i.e. independently cultured and engineered cell aliquots originating from a common neural precursor pool.

In the former case, the promoter was active in cells occupying a terminal position along the neuronogenic sequence and the culture was allowed to age sufficiently for robust Foxg1 protein accumulation within the same cell type where the promoter is active. In the light of these considerations, the interpretation of data obtained in *Foxg1*-OE^pSyn^, type III cultures (Fig. 3G) is straightforward, pointing to a consistent neuronal inhibition of *L1* elements belonging to all families by Foxg1. Remarkably, this inference was corroborated by the results of supplemental *Foxg1* manipulations, both overexpression and loss of function, performed in pure neuronal cultures fully depleted of glial cells by araC supplementation (Fig. 4). In such neocortical neurons, constitutive *Foxg1*-OE reduced the *L1*.orf2 qRT-PCR signal by 25.2±8.7% (*P*<0.046, *n*=4) upon *Gapdh*-normalization (Fig. 4, graph 1), by 33.5±8.7% (*P*<0.025, *n*=4) upon *Rpl10a*-normalization (Fig. 4, graph 2), whereas constitutive *Foxg1*-knockdown (Fig. S2C) increased such signal, by 66.4±21.9% (*P*<0.016, *n*=4) upon *Rpl10a* normalization (Fig. 4, graph 3).

Conversely, in case of type I and type II cultures, the promoters driving the *Foxg1* transgene were active in transient precursor types, within relatively short-lived preparations. Consequently, in such cases, Foxg1 protein accumulation could have taken place in a cell type where the promoter is no longer active, or the available time might have been not sufficient to obtain an appreciable protein upregulation at all. Because of that, *L1*-mRNA dynamics displayed by *Foxg1*-OE type I and type II cultures required further clarification. In this respect, to achieve insights into the actual cell types where Foxg1 upregulation elicited an *L1*-mRNA decline, we (1) quantified the sizes of NSCs, NPs and Ns compartments of differently engineered cultures, (2) profiled each compartment for the distribution of Foxg1 protein cell content, and (3) finally looked for correlative evidence between results of analyses 1 and 2, and cumulative *L1-*mRNA dynamics specific to the corresponding cultures.

**Fig. 5.**
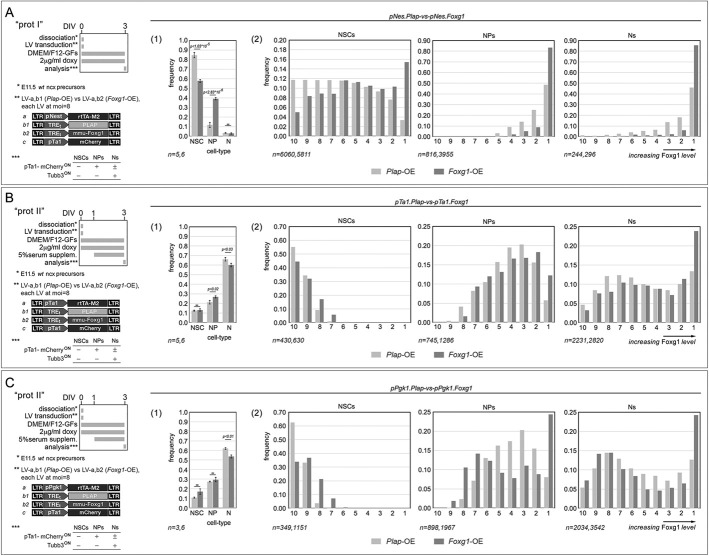
**Quantification of Foxg1 protein levels in distinctive neural precursor types upon *Foxg1* overexpression driven by lentiviral vectors and cell type-specific promoters.** (A-C) Schematics of the protocols and lentiviruses employed are on the left. Graphs show the experimental results. Assays were run in early- (A) and mid- (B,C) neuronogenic cultures, set according to type I and type II protocols, respectively. Cultures overexpressed *Foxg1* or a control transgene (*Plap*). These transgenes were driven by pNes promoter, active in NSCs (A), pTa1 promoter, active in NPs/Ns (B), and pPgk1 promoter, ubiquitously firing (C). NSCs, NPs and Ns were recognized on the basis of their ^pTa1^mCherry^−^/Tubb3^−^, ^pTa1^mCherry^+^/Tubb3^−^, and ^pTa1^mCherry^±^/Tubb3^+^ profiles, respectively. Foxg1 cell content was evaluated by quantitative immunofluorescence. In part 1 graphs, the prevalence of NSCs, NPs and Ns in neural cultures is set according to the different protocols. Error bars indicate s.e.m. Statistical significance of results was evaluated using a one-tailed unpaired *t*-test. *n* is the number of biological replicates, i.e. independently cultured and engineered cell aliquots, originating from pooled, wild-type E11.5 neocortical primordia. In part 2 graphs, the frequencies of NSCs, NPs and Ns falling within distinct Foxg1 expression deciles are shown, i.e. normally equi-numerous bins characterized by decreasing (from 1 to 10) Foxg1 expression levels. *n* is the number of cells profiled, evenly pooled from the biological replicates referred to as for part 1 graphs.

We found that, within early-neuronogenic, type I preparations, pNes-driven *Foxg1* elicited a prominent increase of NPs at the expense of NSCs, while keeping Ns to a minimum. Specifically, if f_X_ indicates the prevalence of X-type cells within the entire cell population, then values were: f_NP_(*Foxg1*-gain of function (GOF)) =39.16±1.14% versus f_NP_(ctrl)=11.93±2.58% [*P*<1.03×10^−5^,*n*=6 (*Foxg1*-OE), *n*=5 (ctrl)], and f_NSC_(*Foxg1*-GOF)=57.81±1.53% versus f_NSC_(ctrl)=84.58±2.81% [*P*<2.83×10^−6^, *n*=6 (*Foxg1*-OE), *n*=5 (ctrl)] (Fig. 5A, part 1).

Moreover, we found that, within the same preparations, pNes-driven *Foxg1* specifically increased the frequency of both NSCs and NPs expressing Foxg1 at the highest levels (i.e. falling within the first decile); however, such an effect was far more prominent in the case of NPs than for NSCs. Specifically, if ^X^f_dec1_ is the fraction of X-type cells falling in decile 1, then values were: ^NSC^f_dec1_(ctrl)=3.4% versus ^NSC^f_dec1_(*Foxg1*-OE)=15.4%, and ^NP^f_dec1_(ctrl)=48.5% versus ^NP^f_dec1_(*Foxg1*-OE)= 83.3% (Fig. 5A, part 2). In this way, among cells moving to the first-decile upon *Foxg1*-OE, (a) more than 4/5 belonged to the NP compartment and (b) fewer than 1/5 belonged to the NSC compartment. In fact, (a) Δf_NP*dec1_=f_NP_(*Foxg1*-OE)×^NP^f_dec1_(*Foxg1*-OE)−f_NP_(ctrl)×^NP^f_dec1_(ctrl)=0.392×0.832−0.119×0.485=0.268), and (b) Δf_NSC*dec1_=f_NSC_(*Foxg1*-OE)×^NSC^f_dec1_(*Foxg1*-OE)−f_NSC_(ctrl)×^NSC^f_dec1_(ctrl)=0.578×0.154−0.846×0.034=0.060.

All that suggests that, within early neuronogenic, type I, cultures, the robust *L1*-mRNA downregulation evoked by *^pNes^Foxg1*-OE (Fig. 3B) mostly occurred in NPs, and the contribution of NSCs to this phenomenon was marginal, if any (Fig. 7, row 1).

Next, we performed a similar analysis of mid-neuronogenic type II, preparations harboring the pTα1- and pPgk1-driven *Foxg1* transgenes, which exhibited the strongest impact on *L1*-mRNA dynamics (Fig. 3D-F). These transgenes altered culture compartments sizes only marginally, both eliciting a moderate shrinkage of the neuronal compartment [from 66.1±1.9% to 60.0±1.8%, with *P*<0.03 and *n*=5 (ctrl), *n*=6 (*Foxg1*-OE) for pTα1, and 62.0±0.9% to 53.6±1.6%, with *P*<0.01 and *n*=3 (ctrl), *n*=6 (*Foxg1*-OE) for pPgk1] (Fig. 5B, part 1; Fig. 5C, part 1). Intriguingly, while similarly perturbing neuronal Foxg1 expression levels, these transgenes distorted Foxg1 protein distribution in NPs according to different patterns. Specifically, the NP fraction ‘moving’ to the first expression decile upon *Foxg1*-OE increased much more in *^pPgk1^Foxg1*-OE cultures (0.244−0.080=0.164) than in *^pTa1^Foxg1*-OE cultures (0.122−0.058=0.064) (Fig. 5B, part 2; Fig. 5C, part 2). Taking into account the stronger *L1* inhibition occurring in *^pPgk1^Foxg1*-OE compared with *^pTa1^Foxg1*-OE cultures (Fig. 3D,E), this scenario suggests that downregulation of *L1*-mRNA detected in *Foxg1*-OE mid-neuronogenic cultures may have primarily occurred in NPs (Fig. 7, row 2). In conclusion, results of our *Foxg1*-OE assays indicate a negative impact of Foxg1 on *L1*-mRNA expression, both in NPs and Ns (Fig. 7), and mirror phenotypes displayed by *Foxg1*-LOF and -OE cultures further suggest that Foxg1 physiologically tunes these levels.

### Mechanisms underlying Foxg1-mediated control of *L1* transcription

**Fig. 6.**
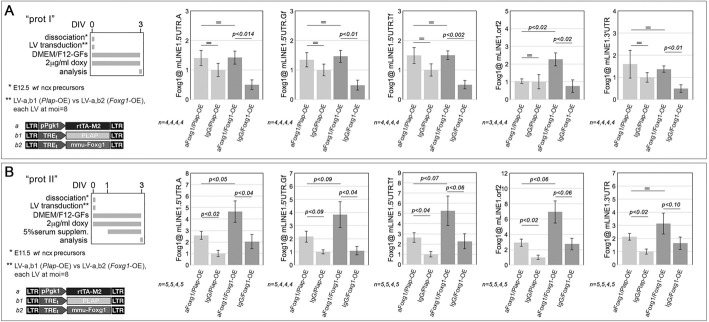
**Chromatin immunoprecipitation profiling of Foxg1 protein enrichment at L1 elements.** (A,B) Schematics of the protocols and lentiviral vectors employed are on the left. Graphs show results from early- (A) and mid- (B) neuronogenic cultures, set according to type I (A) and II (B) protocols. Cultures constitutively overexpressing *Foxg1* (*Foxg1*-OE) or a *Plap*-OE control) are driven by the pPgk1 promoter. Chromatin immunoprecipitation was performed using anti-Foxg1 antibody and control IgG. PCR quantitation of pan-*L1* diagnostic amplicons L1.orf2 and L1.3′UTR, and family-specific L1.5′UTR amplicons .A, .Gf and .Tf is shown. Results are normalized against input chromatin. Error bars indicate s.e.m. Statistical significance of results was evaluated using a one-tailed unpaired *t*-test. *n*=number of biological replicates, i.e. independently cultured and engineered cell aliquots originating from a common neural precursor pool.

**Fig. 7.**
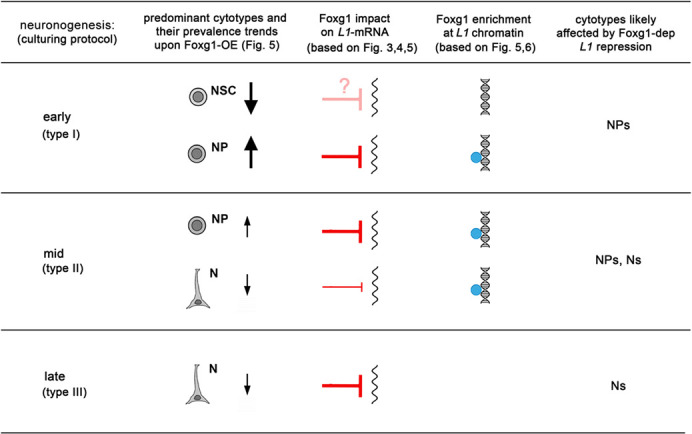
**Foxg1 inhibition of *L1* expression within the pallial neuronogenic lineage.** Tentative cell type-specific unfolding of Foxg1 control of *L1* expression, based on integrated critical evaluation of *L1*-mRNA-qRTPCR (Figs 3,4), Foxg1-qIF-qRTPCR (Fig. 5) and αFoxg1-ChIPqPCR (Fig. 6) results.

Foxg1 is mostly recognized to act as a transcriptional repressor (Falcone et al., 2019; Seoane et al., 2004). We wondered whether this also specifically applies to *L1* retrotransposons. To address this, we established early (‘prot I’-type) neuronogenic cultures, both wild type (*Plap*-OE) and overexpressing Foxg1 (*Foxg1*-OE), and compared Foxg1 enrichment at their *L1* loci against IgG controls, by chromatin immuno-precipitation/quantitative polymerase chain reaction (ChIP)-qPCR. We found that this enrichment was barely detectable in *Plap*-OE cultures and, conversely, was statistically significant at all diagnostic amplicons in *Foxg1*-OE preparations (*P_5_*_′*UTR.A*_<0.014, *P_5_*_′*UTR.Gf*_<0.01, *P_5_*_′*UTR.Tf*_<0.002, *P_orf2_*<0.02, *P_3_*_′*UTR*_<0.01, with *n*=4) (Fig. 6A). Considering the predominance of NSCs in early (prot I-type) *Plap*-OE cultures and their substantial conversion into NPs induced by *Foxg1*-OE (as depicted in Fig. 5A, part 1), ChIP results shown in Fig. 6A may reflect selective Foxg1 recruitment at *L1* loci in NPs, but not in NSCs (Fig. 7, part 1).

Subsequently, we ran similar assays on chromatin prepared from mid-neuronogenic (‘prot II’-type) cultures. In this instance, a clear Foxg1 enrichment was detectable at almost all diagnostic amplicons in *Plap*-OE controls [*P_5_*_′*UTR.A*_<0.02, *P_5_*_′*UTR.Gf*_<0.09, *P_5_*_′*UTR.Tf*_<0.04, *P_orf2_*<0.02 and *P_3_*_′*UTR*_<0.02, with *n*=5 (aFoxg1-IP), *n*≥4 (IgG-IP)] (Fig. 6B). Coupled with the high prevalence of NPs and Ns in all prot II-type cultures (as illustrated in Fig. 5C, part 1), this observation points to Foxg1 binding to *L1* loci in NPs and/or Ns (Fig. 7).

**Fig. 8.**
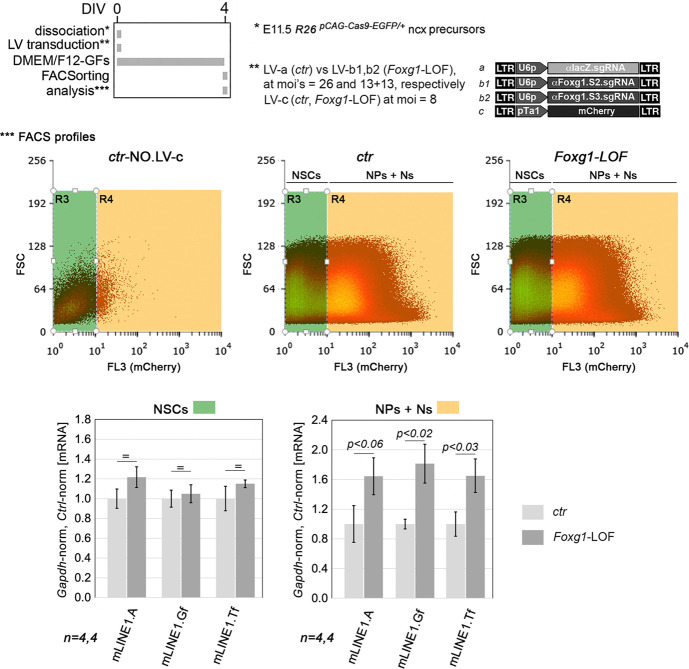
**Assessing *mL1*-mRNA dynamics in *Foxg1* loss-of-function versus control, and NSC- and neuronogenic (NP+N)-sorted populations.** The experimental protocol (top), with details of neocortical (ncx) precursors and lentiviral vectors employed. (Middle) FSC/FL3 distributions of FACsorted cells. Foxg1 was constitutively knocked down by a genetically encoded Cas9 nuclease programmed by two sgRNAs (aFoxg1.S2 and aFoxg1.S3). Non-NSCs, neuronogenic committed cells [including NPs (neuronal progenitors) and neurons (Ns)] and postmitotic neurons were labelled by a lentiviral mCherry transgene driven by the tubulin-a1 promoter (pTa1). A stringent R3 gate, based on the distribution of mCherry-untransduced cells, was applied for clean isolation of NSCs. Graphs show qRT-PCR profiling of sorted cells, normalized against *Gapdh* and further normalized against controls. Error bars indicate s.e.m. Statistical significance of results was evaluated using a one-tailed unpaired *t*-test. *n* is the number of biological replicates, i.e. independently cultured and engineered cell aliquots, originating from pooled *R26^pCAG-Cas9-EGFP/+^* E11.5 neocortical primordia.

In conclusion, both quantification of *L1*-mRNA levels and measurement of Foxg1 protein recruitment to *L1* loci, in control and *Foxg1*-OE cultures, suggested us that Foxg1-driven control over L1 transcription should specifically occur in NPs and Ns. To confirm this, we transduced E11.5 neopallial precursors – either made loss of function for *Foxg1* or left unaltered – with a pTa1-mCherry transgene, driving selective mCherry expression in committed neuronogenic progenitors and their post-mitotic progenies (Brancaccio et al., 2010). Four days later, we dissociated the resulting neurospheres, sorted single cells based on red fluorescence intensity, and quantified *L1* transcripts in mCherry^+^ and mCherry^−^ fractions. It turned out that CRISPR/Cas9-mediated *Foxg1* knockdown did not affect *L1*-mRNA levels in mCherry^−^ NSCs, while inducing a significant upregulation trend of them in mCherry^+^ NPs and Ns [+64.29±24.89% (*P*<0.06), +81.31±26.20% (*P*<0.02) and +64.99±22.54% (*P*<0.03), with *n*=4, for *L1.5*′*UTR.A*, *L1.5*′*UTR.Gf*, and *L1.5*′*UTR.Tf* amplicons, respectively] (Fig. 8). In essence, although significant in NPs and Ns, physiological Foxg1 contribution to *L1* repression is negligible in NSCs, as expected.

Several studies have underscored the significance of various epigenetic marks in tightly regulating L1 transcription (Bulut-Karslioglu et al., 2014; Day et al., 2010; Guler et al., 2017; He et al., 2019; Kim et al., 2014; Muotri et al., 2010; Protasova et al., 2021; Rangasamy, 2013). Additionally, inspection of the public Biogrid database (https://thebiogrid.org/108580/summary/homo-sapiens/foxg1.html) revealed that Foxg1 physically interacts with key effectors modulating the epigenetic chromatin landscape, including histone deacetylase 2 (HDAC2), lysine-specific demethylase 5B (KDM5B) and lysine-specific demethylase 1A (KDM1A). Thus, Foxg1 might influence *L1*-mRNA levels by modulating the epigenetic state of *L1* chromatin.

**Fig. 9.**
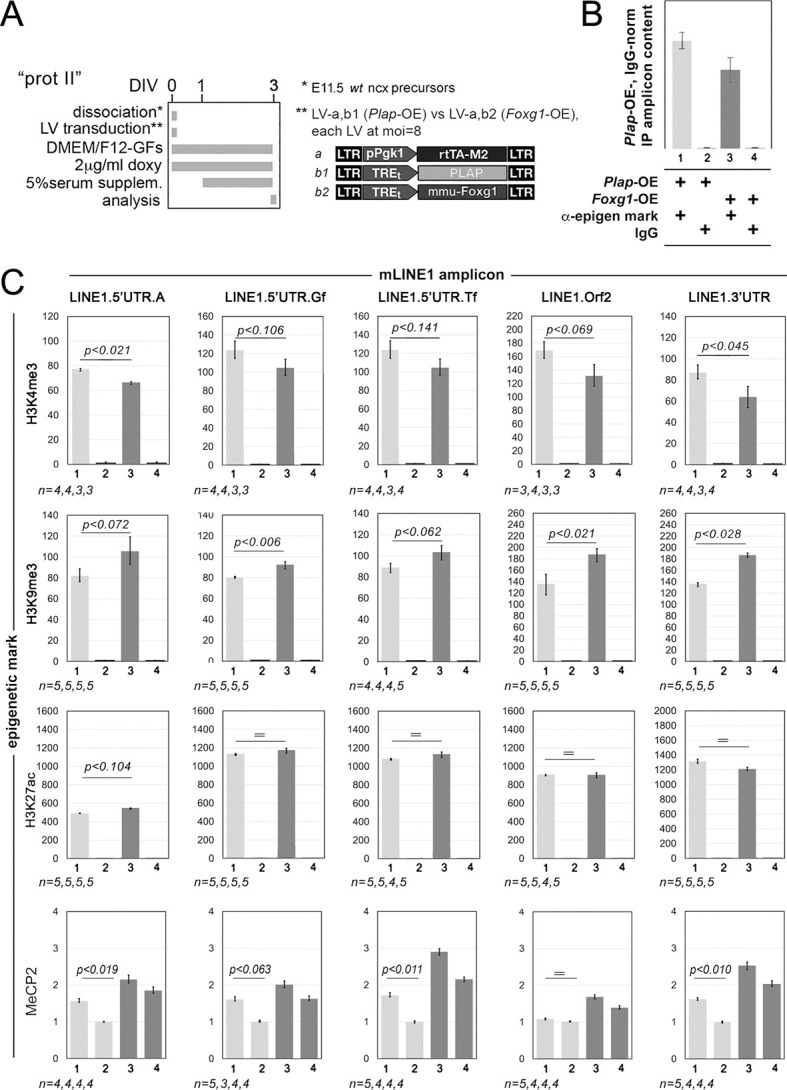
**Chromatin immunoprecipitation profiling of H3K4me3, H3K9me3, H3K27ac and MeCP2 enrichment at *L1* elements.** (A) Schematics of the protocols and lentiviral vectors employed. (B) Schematic to explain samples 1-4 in the graphs in C. (C) Chromatin immunoprecipitation profiling results. Analyses run in mid-neuronogenic cultures, set according to a type II protocol. Cultures constitutively overexpressing *Foxg1* (or a *Plap* control), driven by the pPgk1 promoter. Chromatin immunoprecipitation was performed using anti-H3K4me3, anti-H3K9me3, anti-H3K27ac and anti-MeCP2 antibodies, and their isotypic IgG controls. PCR quantitation of pan-*L1* diagnostic amplicons *L1*.orf2 and *L1*.3'UTR, and family-specific L1.5'UTR amplicons .A, .Gf and .Tf. Results are normalized to chromatin input and Plap-OE/IgG controls. Error bars indicate s.e.m. Statistical evaluation of results by *t*-test (one-tail, unpaired). *n*=number of biological replicates, i.e. independently cultured and engineered cell aliquots originating from a common neural precursor pool.

To explore this, we evaluated chromatin extracted from mid-neuronogenic cultures of wild type and *Foxg1*-OE configuration for its enrichment at *L1* loci for a number of key epigenetic markers: H3K4me3, H3K9me3, H3K27ac and MeCP2 (Fig. 9A,B). We observed a remarkable enrichment for H3K4me3, H3K9me3, H3K27ac at all diagnostic amplicons analyzed, *L1*.5′UTR.A, .Gf, .Tf, .orf2 and .3′UTR, irrespective of culture genotype (about 60- to 1200-fold over IgG controls). Conversely, enrichment for MeCP2 over these controls was barely appreciable (<2-fold). Furthermore, enrichment for H3K4me3 showed a decreasing trend in *Foxg1*-OE compared with *Plap*-OE cultures [*P_5_*_′*UTR.A*_<0.021, *P_5_*_′*UTR.Gf*_<0.106, *P_5_*_′*UTR.Tf*_<0.141, *P_orf2_*<0.069 and *P_3_*_′*UTR*_<0.045, with *n*=3 (*Foxg1*-OE) and *n*≥3 (*Plap*-OE)]. Conversely, an opposite trend was evident for H3K9me3 (*P_5_*_′*UTR.A*_<0.072, *P_5_*_′*UTR.Gf*_<0.006, *P_5_*_′*UTR.Tf*_<0.062, *P_orf2_*<0.021 and *P_3_*_′*UTR*_<0.028, with *n*≥4 (*Foxg1*-OE) and *n*≥4 (*Plap*-OE) (Fig. 9C).

Overall, these results support the hypothesis that Foxg1-mediated modulation of *L1* transcription involves pervasive changes in the epigenetic state of these elements, namely a decrease in transcription-promoting H3K4me3 marks and an increase in heterochomatic H3K9me3 marks. Additionally, the high levels of H3K27ac observed in both controls and *Foxg1*-OE samples suggest a transient bivalent state of chromatin, capable of both silencing and transcription (He et al., 2019), whereas the low MeCP2 enrichment at mid-neuronogenic stages likely reflects relatively low expression of this protein (Diez-Roux et al., 2011).

**Fig. 10.**
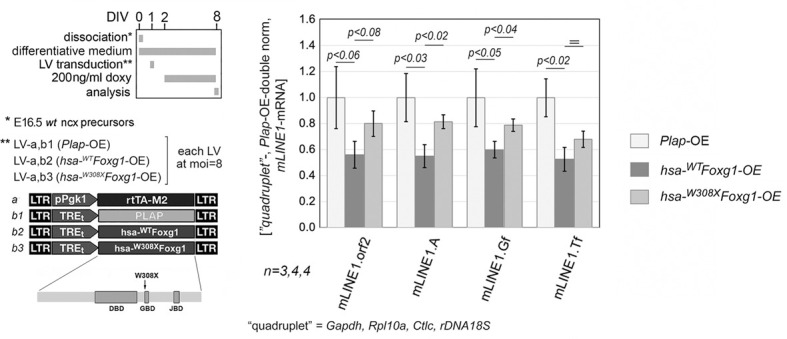
**Modulation of *L1*-mRNA levels in primary neuron-enriched murine cultures overexpressing the human, wild-type and mutant *hsa-^WT^Foxg1* and *hsa-^W308X^Foxg1* alleles.** Schematics of the protocols and lentiviral vectors employed are illustrated on the left. Transgenes were driven by the constitutively firing pPgk1 promoter. The mutant allele (W308X) sequentially encodes a prematurely truncated protein, including the DNA-binding domain (DBD), but not the Groucho- and Jarid-binding domains (GBD and JBD, respectively). RT-PCR quantitation of pan-*L1* diagnostic amplicon *L1*.orf2, and family-specific amplicons *L1*.A, *L1*.Gf and *L1*.Tf, in neural cultures set according to a type II protocol. Data are double normalized against a gene quadruplet (*Gapdh*, *Rpl10a*, *Cltc* and *rDNA 18S*) and control values. Error bars indicate s.e.m. Statistical significance of results was evaluated using a one-tailed unpaired *t*-test. *n* is the number of biological replicates, i.e. independently cultured and engineered cell aliquots, originating from pooled wild-type E16.5 neocortical primordia.

Finally, to further elucidate the mechanisms mediating the impact of Foxg1 on *L1* transcription, we took advantage of the neuropathogenic *FOXG1^W308X^* allele (Frisari et al., 2022) encoding a prematurely truncated protein, and lacking the binding domains for the Groucho/Tle co-repressor and the *KDM5B*-encoded JARID1B H3K4me2/3-demethylase (Fig. 10). Delivered to neuron-enriched cultures as a Tet^ON^-driven transgene, *FOXG1^W308X^* led to a reduction of *L1*-mRNA levels, less pronounced compared with *FOXG1^WT^*, −19.93±9.85% versus −43.94±10.39% (*n*=4), −18.47±5.27% versus −45.01±8.83% (*P*<0.021; *n*=4), −21.11±4.72% versus −40.03±6.37% (*P*<0.038; *n*=4) and −31.94±6.24% versus −47.37±9.15% (*n*=4), as evaluated at diagnostic amplicons orf2, 5′UTR.A, 5′UTR.Gf and 5′UTR.Tf, respectively (Fig. 10). A consistent pattern emerged from comparisons of earlier, ‘mid-neuronogenic’, cultures, alternatively overexpressing the two FOXG1 alleles (Fig. S3 and Fig. 3D), suggesting that JARID1B and/or Groucho/Tle contribute to *Foxg1*-dependent *L1* repression.

### Temporal progression of pallial *L1* DNA copy-number

**Fig. 11.**
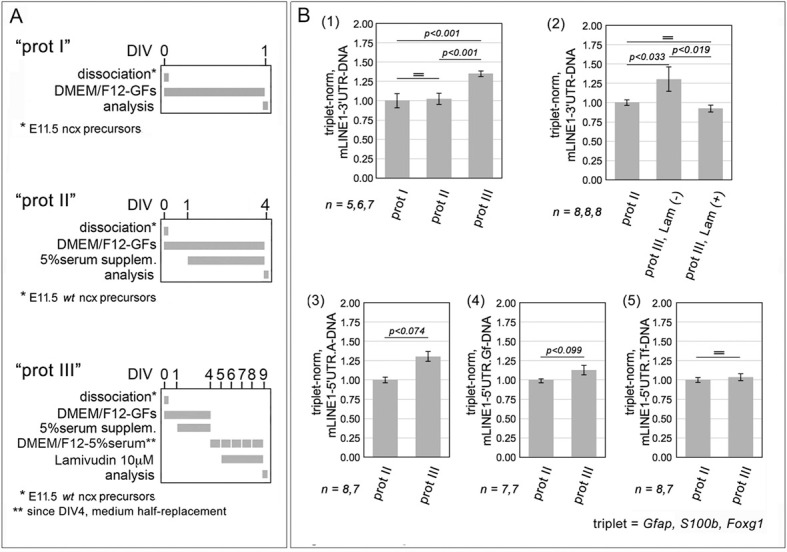
***L1* DNA copy number variations (CNVs) in early- mid- and late-neuronogenic murine pallial cultures.** (A,B) Schematics of the protocols (A) and results (B). Early- mid- and late-neuronogenic cultures set by means of type I, II and III protocols, respectively. DNA extraction was performed by high PK (part 1) and very high PK (parts 2-5) procedures (see Materials and Methods). DNA CNVs were assessed by quantitative PCR, followed by normalization against endogenous *Gfap*, *S100b* and *Foxg1* (a gene triplet). Part 1 shows the total *L1* copy number detectable in late- and mid-normalized cultures versus early-neuronogenic cultures. Part 2 shows the suppression of *L1* copy number variation, which normally occurs between mid- and late-neuronogenic cultures, elicited by the pan-RT inhibitor lamivudine (also known as 3T3). Finally, parts 3-5 (controls) show comparisons of family-specific DNA copy numbers, as detected by 5′UTR.A, 5′UTR.Gf and 5′UTR.Tf diagnostic amplicons. Errors bars indicate s.e.m. Statistical significance of results was evaluated using a one-tailed unpaired *t*-test. *n* is the number of biological replicates, i.e. independently cultured and engineered cell aliquots, originating from pooled, wild-type E11.5 neocortical primordia.

We wondered whether, in addition to inhibiting *L1* transcription of *L1* elements, Foxg1 might further impact their DNA copy number. To obtain preliminary information about natural dynamics of *L1* DNA within the developing embryonic pallium, we scored early-, mid- and late-neuronogenic cultures for their cumulative *L1* copy number (Fig. 11A). For this purpose, we relied on the diagnostic 3'UTR amplicon (Fig. S1, Table S1), present in all *L1* repeats, including the prevailingly 5′ truncated elements originating from somatic retrotransposition (Babushok et al., 2006). We found *L1* copy number did not change across early- and mid-neuronogenic cultures, whereas it was increased by 35.08±3.65% [*P*<0.001, *n*=5 (early-neuronogenic), *n*=7 (late-neuronogenic)] in late-neuronogenic cultures (Fig. 11B, part 1).

These results were obtained on DNA prepared by a dedicated sample digestion procedure (‘high PK’), aimed at extracting DNA with comparable efficacy regardless of the compaction state of chromatin. [Fulfillment of this requirement had been previously tested, by quantifying an X-chromosomal (lyonizable) *Mecp2* amplicon in DNA extracted from female and male tissues, and normalizing it against an autosomal amplicon (*Gfap*). This gave a normalized, female-to-male *Mecp2* signal ratio, equaling 1.54±0.18 [with *P*_♂-♀_<0.053, *n=*3 (♂), *n*=2 (♀) (Fig. S5, part 1).] To strengthen the results in Fig. 11B, part 1, we repeated the quantification of pallial *L1* content upon replacing the high PK protocol with a further improved version of it (‘very high PK’). [With this latter protocol the female-to-male *Mecp2* signal ratio rose to 2.25±0.45 [*P*_♂-♀_<0.016, *n*=3 (♂), *n*=2 (♀)] and a similar 2.22±0.10 ratio was also obtained for an alternative X-chromosomal gene, *Cdkl5* (*P*_♂-♀_<0.001, *n*=3) (Fig. S5, parts 2 and 3).] Moreover, as a control, we included in this last assay late-neuronogenic cultures pre-treated by chronic lamivudine, an established inhibitor of retro-transcription. Very high PK samples substantially replicated the outcome of high PK samples, with *L1* copy number increased in late-neuronogenic cultures by 1.31±0.16-fold compared with their mid-neuronogenic counterparts (*P*<0.04, *n*=8). Remarkably, this increase was fully suppressed by lamivudine (Fig. 11B, part 2).

As mentioned above, assays referred to in Fig. 11B, parts 1 and 2 were based on the 3′UTR diagnostic amplicon. As a further control, we also quantified *L1*-DNA in mid- and late neuronogenic cultures by means of family-specific 5'UTR amplicons (Fig. S1, Table S1). As expected, no relevant changes were found (Fig. 11B, parts 3, 4 and 5), except an increasing trend in 5′UTR.A (Fig. 11B, part 3), possibly reflecting differential somatic reverse transcription failure in distinct *L1* families.

**Fig. 12.**
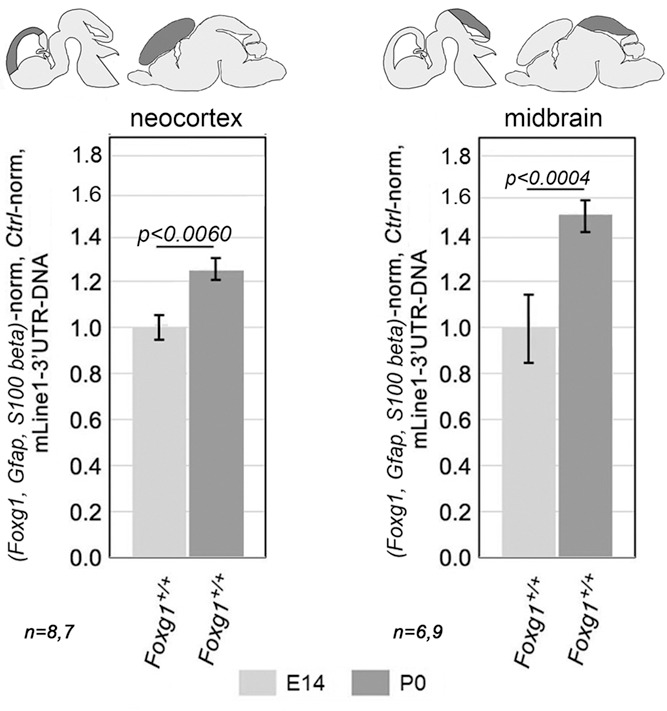
***L1* DNA copy numbers in the neocortex and tectum of wild-type, mid-neuronogenic and perinatal mice.** PCR quantitation of the pan-*L1* diagnostic amplicon *L1*.3'UTR. Data are normalized against the *Foxg1*, *Gfap* and *Nfia* gene triplet. Error bars indicate s.e.m. Statistical significance of results was evaluated using a one-tailed unpaired *t*-test. *n* is the number of biological replicates, i.e. neocortices taken from distinct pups.

Finally, to validate the dynamics of *L1*-DNA observed in mid- versus late-neuronogenic cultures, we compared *L1*-DNA content in neocortices dissected from E14.5 versus P0 wild-type mice. As expected, the latter exceeded the former by 23.8±4.7% [*P*<0.0060; *n*=8 (E14.5), *n*=7 (P0)] (Fig. 12), corroborating our previous findings. Intriguingly, an increase of *L1*-DNA content over the same time interval was also detectable in the mesencephalic tectum, where its amplitude was even larger [+54.0±8.1%, with *P*<0.0004 and *n*=6 (E14.5), *n*=9 (P0)] (Fig. 12).

### The impact of Foxg1 on *L1*.DNA copy numbers

**Fig. 13.**
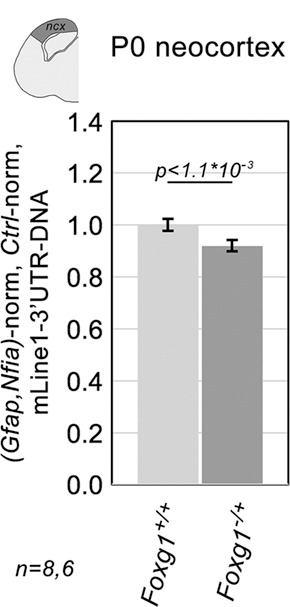
***L1* DNA copy numbers in the neocortex of *Foxg1^−/+^* mouse neonates and controls.** PCR quantitation of the pan-*L1* diagnostic amplicon *L1*.3'UTR. Data are double normalized against the *Gfap* and *Nfia* gene doublet, and wild-type controls. Error bars indicate s.e.m. Statistical significance of results was evaluated using a two-tailed unpaired *t*-test. *n* is the number of biological replicates, i.e. neocortices taken from distinct pups.

We have shown that *L1* copy number increases during neocortical neuronogenesis progression. To further investigate the role (if any) of *Foxg1* in this process, we compared *L1* DNA content in neocortices of *Foxg1^−/+^* neonates and their littermate wild-type controls. When normalized against *Gfap* and *Nfia*, such content turned out to be decreased in *Foxg1* loss-of-function samples by 7.50±0.85% [*P*<10^−3^, *n*=8 (*Foxg1*^*+/+*^), *n*=6 (*Foxg1*^*−/+*^)], compared with controls (Fig. 13). Notably, this variation accounts for approximately one-third of the increment in neocortical *L1* copies detectable over the same time interval in wild-type mice (Fig. 12). Moreover, this decrease occurred in mutants characterized by *Foxg1*-mRNA levels reduced by only 33.59±6.17% [*P*<0.02, *n*=8 (*Foxg1*^*−/+*^), *n*=7 (*Foxg1*^*+/+*^)] compared with wild-type controls (Table S2).

**Fig. 14.**
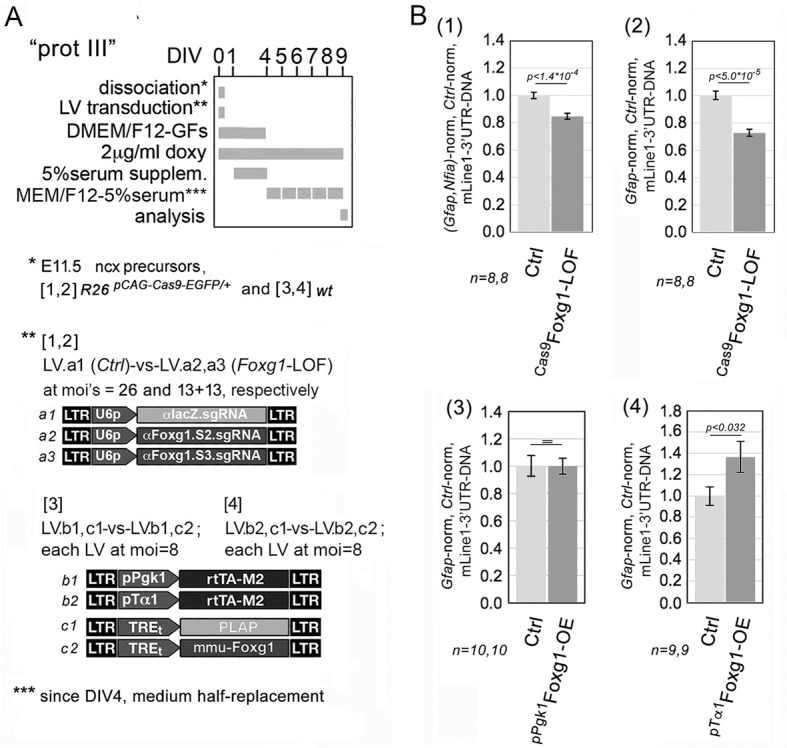
**Impact of *Foxg1*-knockdown and *Foxg1*-overexpression on *L1* DNA copy numbers in late-neuronogenic pallial cultures.** (A) Schematics of the protocols (with lentiviruses employed). CRISPR-Cas9 machinery was driven by constitutively active U6p (sgRNAs) and R26/pCAG (Cas9) promoters; the Foxg1 transgene is led by constitutive Pgk1p and NP/N-restricted pTa1. Neural cultures were set according to a type III protocol. (B) Assessment of total L1 copies by quantitation of the *L1*.3'UTR amplicon. Data are double-normalized against *Gfap* (or the *Gfap* and *Nfia* gene doublet) and control values. Error bars indicate s.e.m. Statistical significance of results was evaluated using a one-tailed unpaired *t*-test. *n* is the number of biological replicates, i.e. independently cultured and engineered cell aliquots, originating from pooled *R26^pCAG-Cas9-EGFP/+^* (1,2) and wild-type (3,4) E11.5 neocortical primordia.

To corroborate these findings, we repeated the evaluation of *L1* copy number in primary, late-neuronogenic cultures manipulated by CRISPR-Cas9 technology, which allowed us to achieve a more pronounced *Foxg1* downregulation, by 65.37±2.68% [*P*<10^−7^, *n*=8 (^Cas9^*Foxg1*-LOF), *n*=8 (ctrl)] (Table S2). Remarkably, in this case, *L1* copy number was reduced by 15.23±2.19% (upon normalization against *Gfap* and *Nfia*, with *P*<10^−3^, *n*=8 (^Cas9^*Foxg1*-LOF), *n*=8 (ctrl)] (Fig. 14B, part 1), corresponding to about two-thirds of the ‘physiological’ increment mentioned above (Fig. 12).

To achieve a more comprehensive understanding of the role of Foxg1 in tuning *L1* copy number, we overexpressed it in late-neuronogenic cultures, under the control of pTa1 and pPgk1 promoters, and evaluated the impact of these manipulations on *L1*-DNA content. As expected, we found that *L1*-DNA content was increased by ‘pTa1-driven *Foxg1*’, by 37.11±16.35%, with *P*<0.03 and *n*=9 (ctrl), *n*=9 (^pTa1^*Foxg1*-OE) (Fig. 14B, part 4). Conversely, no *L1*-DNA increase was elicited by ‘pPgk1-driven *Foxg1*’ (Fig. 14B, part 3), possibly due to the stronger *L1*-mRNA downregulation triggered by this transgene compared with its pTa1 counterpart (Fig. 3D,E). These results collectively indicate that Foxg1 plays a crucial role in *L1*-DNA amplification and its fine physiological tuning. Notably, such amplification-promoting activity must be particularly robust, as it emerged despite the concurrent *Foxg1*-induced downregulation of *L1*-mRNA, which is the template from which new *L1* DNA is generated.

### Mechanisms underlying Foxg1 impact on *L1* copy number

We wondered how Foxg1 could impact *L1* DNA content and considered two possible scenarios: (1) it acts indirectly, as a ‘professional transcription factor’ that modulates the transcription of genes encoding key effectors involved in synthesis and/or degradation of new somatic *L1* copies; or (2) it directly regulates these processes, through physical interaction with factors implicated in them and/or with *L1*-mRNA.

To investigate (1), we inspected a database of genes mis-regulated upon Foxg1-OE in neocortical neuronal cultures (Artimagnella and Mallamaci, 2020). We found that mRNA encoding Apobec1, an inhibitor of *L1* retro-transposition (Ikeda et al., 2011), is halved in *Foxg1*-OE samples (Table S4A), which suggests Foxg1 might promote retro-transposition, by mitigating such inhibition.

**Fig. 15.**
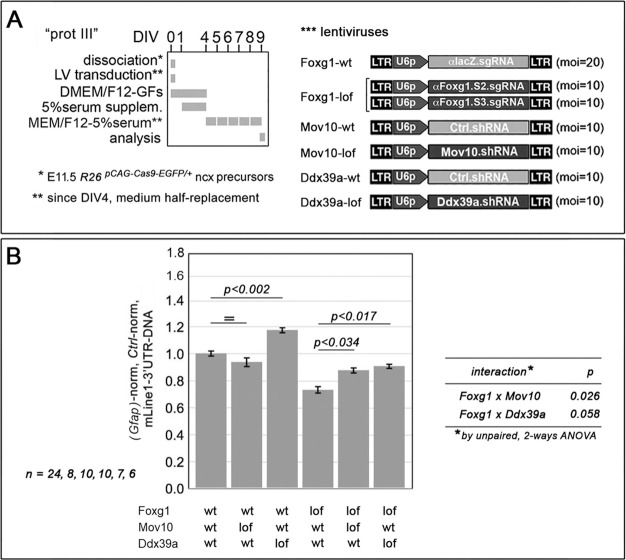
**Functional interaction among *Foxg1* and helicase genes *Mov10* and Ddx39a in the modulation of L1 copy number.** (A) Schematics of the protocols (with lentiviruses employed). (B) The impact exerted by *Mov10* and *Ddx39a* downregulation in *Foxg1*-wild-type (wt) and *Foxg1*-loss-of-function (LOF) environments on L1 copy number, as evaluated in late-neuronogenic cultures by qPCR. Data are double-normalized against *Gfap* and control values. *n* is the number of biological replicates, i.e. independently cultured and engineered preparations, originating from a common neural cell pool. Statistical significance of results was evaluated using a one-tailed unpaired *t*-test and by an unpaired, two-way ANOVA. Errors bars indicate s.e.m.

To investigate (2), we interrogated the public Biogrid database for Foxg1 interactors implicated in retro-transposition control and found two well-known antagonizers of *L1* retro-transposition: Mov10 and Ddx39a (Goodier et al., 2012) (Table S4B). We co-manipulated expression levels of each of them alongside *Foxg1* in late-neuronogenic cell preparations, and evaluated the impact of such manipulations on *L1*-DNA content (Fig. 15A). Intriguingly, in a sensitized *Foxg1* loss-of-function environment, ‘wild-type’ levels of both *Mov10* and *Ddx39a* led to statistically significant decreases in *L1*-DNA copy number compared with their knockdown counterparts (−16±3% with *P*<0.035, *n*=7; −18±3% with *P*<0.018, *n*=6, respectively). Conversely, in a *Foxg1* wild-type environment, a decrease was detectable only in *Ddx39a* wild-type compared with *Ddx39a* knockdown samples (−15±3% with *P*<0.015, *n*=10). Notably, two-way ANOVA of results indicated statistical interaction among *Foxg1* and both *Mov10* (*P*<0.026) and *Ddx39a* (*P*<0.058) variables (Fig. 15B), pointing to a likely functional interaction among Foxg1 and the helicases encoded by these two genes.

**Fig. 16.**
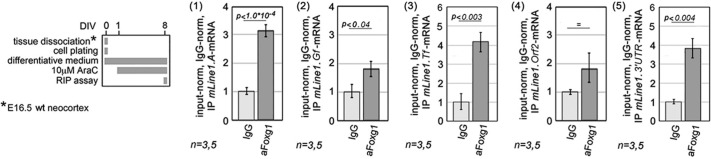
**Foxg1-protein/*L1*-mRNA interaction in neocortical neurons, as evaluated by a RNA immunoprecipitation-quantitative PCR (qRIP-PCR) assay.** A schematic of the protocol is on the left. Diagnostic amplicons used were L1.5′UTR.A (1), L1.5′UTR.Gf (2), L1.5'UTR.Tf (3), L1.orf2 (4) and L1.3'UTR (5). Results are double normalized against input-RNA and IgG-IP samples. Throughout figure, *n* is the number of biological replicates, i.e. independently cultured and engineered preparations, originating from a common neural cell pool. Statistical evaluation of results performed by a one-tailed unpaired *t*-test. Errors bars indicate s.e.m.

Furthermore, we reasoned that Foxg1 could counteract Mov10 and Ddx39a, by preventing them from interacting with *L1*-mRNA. This might be achieved by chelating these helicases and/or shielding their *L1*-RNA interactor. Although the former phenomenon has been previously documented (Li et al., 2015), to assess the latter we ran a set of RNA-immunoprecipitation (RIP) assays, with which we quantified 5′UTR, orf2 and 3′UTR *L1* diagnostic amplicons in anti-Foxg1-immunoprecipitated RNA (Fig. 16). Consistent with our prediction, we found a robust enrichment of Foxg1 at both 5′ and 3′ ends of L1-mRNA. Normalized against IgG controls, this enrichment was 3.11±0.20-fold at L1.5′UTR.A (*P*<10^−4^, *n*=5,3), 1.85±0.28-fold at L1.5′UTR.Gf [*P*<0.043, *n*=5 (anti-Foxg1), *n*=3 (lgG)], 4.13±0.51-fold at L1.5′UTR.Tf [*P*<0.003, *n*=5 (anti-Foxg1), *n*=3 (lgG)] and 3.89±0.55-fold at L1.3′UTR [*P*<0.004,*n*=5 (anti-Foxg1), *n*=3 (lgG)] (Fig. 16, parts 1, 2, 3 and 5). Foxg1 enrichment was lower at L1.orf2, where statistical significance was not reached (Fig. 16, part 4).

## DISCUSSION

Foxg1 plays a central role in telencephalic development, exerting a highly pleiotropic control over it. *L1* is a prominent retrotransposon family modulating progression of neocortical histogenesis and contributing to plasticity of neuronal genomic DNA. In this study, we systematically investigated the impact of the former on the biology of the latter within the developing murine neocortex. The main results were as follows.

As suspected, we found that *L1*-mRNA encoded by all three retro-transposition-competent families (A, Gf and Tf) was increased in *Foxg1* loss-of-function mouse neonates compared with wild-type controls (Fig. 1). To model *Foxg1*-dependent *L1* regulation across neuronogenic progression, we first developed an integrated culture set, representing early, mid and late phases of neuronogenesis *in vitro*. This set exhibited a progressive increase in *L1*-mRNA expression, paralleling *in vivo L1*-mRNA dynamics (Fig. 2). We then manipulated Foxg1 levels at different stages of the neuronogenic progression, both raising and lowering, using multiple approaches, taking advantage of distinctive neural cell type-specific promoters (Figs 3,4). We evaluated the impact of these manipulations on the sizes of NSCs, NPs and Ns compartments, and we mapped changes of Foxg1 protein levels to these compartments (Fig. 5). Additionally, we profiled Foxg1 binding to *L1* chromatin at different steps of neuronogenic progression (Fig. 6). Integrated analysis of these results provided us with robust evidence that Foxg1 represses *L1* expression selectively in NPs and Ns (Fig. 7). This repression was confirmed by quantifying *L1*-mRNA in NSCs and NPs+Ns fractions, FACsorted from *Foxg1* loss-of-function preparations (Fig. 8). As expected, we found that such repression was associated with reduced H3K4me3 and increased H3K9me3 marks along the entire retrotransposon (Fig. 9). Furthermore, a prematurely truncated, human neuropathogenic, loss-of-function variant of FOXG1 also downregulated *L1*-mRNA, although to a lesser extent than its ‘healthy’ counterpart (Fig. 10 and Fig. S3).

Before investigating the potential impact of *Foxg1* on *L1*-DNA copy number, we profiled the progression of this number in early-, mid- and late-neuronogenic cultures, and we found that it increased by ∼35% in late cultures, in a retro-transcription-dependent way (Fig. 11). A similar increase was also observed *in vivo*, in neonatal compared with mid-neuronogenic embryonic neocortex, suggesting the former phenomenon to be genuine (Fig. 12). Then, unexpectedly, we discovered that Foxg1 downregulation, both *in vivo* and *in vitro*, resulted in a remarkable dose-dependent reduction in *L1*-DNA content that was up to two-thirds of the natural increase observed *in vivo*. Consistently, mild Foxg1-OE in mid-neuronogenic cultures increased *L1*-DNA, further suggesting that Foxg1 tunes physiological amplification of this DNA (Figs 13,14). We hypothesized that Foxg1 intervention in *L1* retrotranscription might involve two helicases, Mov10 and Ddx39a, that are known to antagonize retrotransposition and physically interact with Foxg1 protein. Interestingly, Foxg1 desensitized neocortical neurons to the activity of these helicases, resulting in increased *L1*-DNA copy number (Fig. 15). Finally, we found that Foxg1 binds to *L1*-mRNA, particularly at its 5′ and 3′ ends (Fig. 16).

As a general methodological note, the interpretation of results originating from gene overexpression assays requires a special caution, because of paradoxical dominant-negative effects potentially evoked by gene product overabundance. In this respect, it is worth mentioning that, in our overexpression assays, *Foxg1* expression gains usually fell below fivefold, at both mRNA and protein levels (Table S2 and Fig. S2). More importantly, phenotypes evoked by Foxg1-OE generally mirrored those elicited by gene knockdown (see, for example, Fig. 3A versus Fig. 3B-G or Fig. 4, part 2 versus Fig. 4, part 3), suggesting that they provide a qualitatively genuine representation of the physiological functions played by Foxg1 in natural contexts.

Concerning the scientific outcome of our study, two main messages emerged from it.

(1) We demonstrate that *L1*-mRNA levels progressively increase as neopallial neuronogenesis goes on, and Foxg1 limits this increase (Figs 3,4). Noticeably, Foxg1 control of *L1*-mRNA applies to all three retrotransposition-competent L1 families (Figs 1,3) and it is achieved by direct Foxg1 binding to *L1* chromatin (Fig. 6), triggering a prominent change in its epigenetic state (Fig. 9).

(2) We document a natural upregulation of pallial *L1*-DNA content occurring during late-intrauterine development, and discover that, albeit associated to an opposing *L1*-mRNA dynamic (Fig. 3), moderate fluctuations in Foxg1 levels around the baseline generally result in colinear variations in such *L1*-DNA content (Figs 13,14, parts 1,2 and 4).

In fact, the increasing progression of *L1*-mRNA levels we documented in the murine neopallial neuronogenic lineage has been reported previously. Indeed, it recalls similar phenomena that take place in human embryonic neocortex (Garza et al., 2023) and adult hippocampus (Muotri et al., 2010). However, the impact exerted by Foxg1 on *L1*-mRNA expression has not been reported previously. Thus, Foxg1 adds to the small transcription factor set known to control *L1* transcription in the CNS (Blaudin De Thé et al., 2018; Kuwabara et al., 2009; Liu et al., 2019; Muotri et al., 2005; Nandi et al., 2016; Sanchez-Luque et al., 2019). In this context, it is similar to Sox2. However, Sox2 has been documented to repress *L1* transcription in adult NSCs (Kuwabara et al., 2009), whereas Foxg1 has been shown to act in embryonic neuronal progenitors and neurons (Figs 7 and 8). Moreover, Sox2 is expressed in the apical compartment of the entire neuraxis (Pevny and Lovell-Badge, 1997), whereas Foxg1 is mainly confined to the telencephalon (Tao and Lai, 1992). To our knowledge, Foxg1 is the first patterning gene proven to limit *L1* expression within a specific domain of the developing mouse brain (Blaudin De Thé et al., 2018; Kuwabara et al., 2009).

The increase of *L1*-DNA content we documented in the developing mouse neocortex has been reported already. Indeed, it is qualitatively and metrically consistent with results of a previous study, run in the perinatal rodent brain (Fontana et al., 2021). However, the impact of Foxg1 on *L1*-DNA copy number has not been reported before. In this respect, we hypothesize that *Foxg1*-mediated control of *L1*-DNA content could take place via two helicases, Mov10 and Ddx39a, which are reported to antagonize *L1* retro-transcription (Goodier et al., 2012) and to interact physically with Foxg1 protein (Li et al., 2015). We rule out transcription as a mediator of this mechanism. In fact, although it resulted in *Mov10*- and *Ddx39a*-mRNA downregulation (by −45.3% and −17.6%, respectively, with *P*adj<0.05; O.A. and A.M., unpublished), *Foxg1* knockdown did not increase *L1*-DNA content, but rather reduced it. Conversely, we noticed that *Foxg1* knockdown made the decline in *L1*-DNA elicited by higher levels of *Mov10* and *Ddx39a* more pronounced (Fig. 15). In addition, we show that Foxg1 protein normally binds to *L1*-mRNA (Fig. 16). Hence, we propose that Foxg1 may ease *L1-*mRNA retro-transcription largely by preventing the interaction among the two helicases and *L1-*mRNA (Goodier et al., 2012), either competitively or due to steric hindrance. Such involvement in retrotranscription control supports a role for Foxg1 in other non-transcriptional metabolic routines, such as post-transcriptional ncRNA processing (Weise et al., 2019), translation (Artimagnella et al., 2022 preprint) and mitochondrial biology (Pancrazi et al., 2015).

Such a bimodal impact of Foxg1 on *L1* biology is remarkable. Mechanistically, it is tempting to speculate that the transient downregulation *Foxg1* physiologically undergoes in newborn pyramids (Miyoshi and Fishell, 2012) may be instrumental in allowing sufficient accumulation of *L1*-mRNA needed for subsequent robust retro-transcription. From a broader perspective, it has been shown and/or suggested that different products of L1 activity (RNA, orf1/2 proteins and DNA) have been evolutionarily hijacked for distinctive aspects of cell physiology and metabolism (Blaudin De Thé et al., 2018; Chow et al., 2010; Madabhushi et al., 2015; Mangoni et al., 2023; Muotri and Gage, 2006), so likely require differential tuning of their doses. Thanks to its bimodal impact on L1 transcription and retrotranscription, Foxg1 might contribute to such complex regulation. Notably, the relationship between *L1*-mRNA and *L1*-DNA levels apparently depends on the type of CNS structure. In contrast to the neocortex, a huge amplification of *L1*-DNA content is achieved within the late-gestational tectum, despite the concomitant downregulation of the underlying *L1*-mRNA level (Figs 2, 12). This might reflect an intrinsically different regulation of L1 biology in telencephalon versus mesencephalon and/or a developmental heterochrony between these two structures.

At the moment, we ignore the functional meaning of *Foxg1*-mediatedcontrol over *L1* biology. Concerning Foxg1-dependent modulation of *L1*-mRNA levels, two considerations may help addressing this issue. On one side, *Foxg1* exerts a multifaceted impact on neocortical histogenesis: (1) stimulating the NSC-to-NP transition (Falcone et al., 2019; Fig. 5A part 1); (2) inhibiting NPs from exiting the cell cycle (Brancaccio et al., 2010); (3) promoting postmitotic neuronal differentiation (Chiola et al., 2019; Frisari et al., 2022; Tigani et al., 2020; Yu et al., 2019; Zhu et al., 2019) and migration (Miyoshi and Fishell, 2012); and (4) antagonizing gliogenesis (Brancaccio et al., 2010; Falcone et al., 2019; Frisari et al., 2022). On the other side, it has been shown that specific ensembles of transposable elements are transcribed concomitantly with the progression of some early histogenetic routines (He et al., 2021). In some cases, such transcription has been proven necessary for the advancement of these routines (Jachowicz et al., 2017; Macfarlan et al., 2012; Percharde et al., 2018). It is tempting to speculate this might also apply to cortical histogenesis. In this respect, shortly after the submission of this manuscript, Mangoni et al. reported an in depth dissection of the complex phenotype originating from *L1*-mRNA knockdown in the developing neocortex (Mangoni et al., 2023). In our study, *L1*-mRNA was dampened via RNAi, by an order of magnitude comparable with that elicited via *Foxg1*-OE (Fig. 3D). Intriguingly, this resulted in a variety of histogenetic anomalies, some of which (such as increased NSC progression to neuronogenesis, impaired neuronal radial migration and decreased astrogenesis) were highly reminiscent of the developmental phenotype evoked by *Foxg1*-OE (Fig. 5A, part 1; Falcone et al., 2019; Miyoshi and Fishell, 2012). This suggests that the response of *L1* to *Foxg1* may contribute to *Foxg1*-driven regulation of these processes.

Regarding the impact of Foxg1 on *L1-*DNA copy number, it has been proposed that somatic retrotransposition may help to diversify the neuronal genome and, therefore, neuronal functional properties (Muotri and Gage, 2006; Singer et al., 2010). By upregulating *L1-*DNA copy number, Foxg1 might enhance this phenomenon.

Finally, beyond its physiological occurrence in the developing rodent embryo, the relationship between *FOXG1* and *L1* elements could be relevant to the etiopathogenesis of the human *FOXG1* syndrome. Deficient FOXG1 activity linked to *FOXG1* hemizygosity or heterozygosity for loss-of-function alleles might lead to *L1*-mRNA upregulation, while supernumerary or gain-of-function *FOXG1* alleles might result in exaggerated *L1*-DNA neo-synthesis. Both scenarios are of potential neuropathogenic interest (Suarez et al., 2018), and early treatment with inhibitors of retrotranscription approved by the United States Food and Drug Administration (New drugs for HIV infection, 1996) might mitigate consequences of *FOXG1* gain-of-function mutations. However, major differences characterize cortical histogenesis and *L1* biology in humans and rodents (Pinson and Huttner, 2021; Rosser and An, 2012). For these reasons, these issues deserve further in-depth investigations.

## MATERIALS AND METHODS

### Animal handling

Animal handling and subsequent procedures were carried out in accordance with European and Italian laws [European Parliament and Council Directive of 22 September 2010 (2010/63/EU); Italian Government Decree of 4 March 2014, n° 26]. Experimental protocols were approved by SISSA OpBA (Institutional SISSA Committee for Animal Care).

Embryos and animals were generated at the SISSA mouse facility, as follows: wild-type mice were generated by breeding CD1 parents, purchased from Envigo Laboratories, Italy; *Foxg1*^+/−^ mice (and their wild-type controls) were generated by breeding CD1-backcrossed *Foxg1*^+/−^ males (Hébert and McConnell, 2000) to wild-type CD1 females; *Rosa26^pCAG-Cas9-2P2-Egfp)/+^* mice were generated by breeding *Rosa26^pCAG-Cas9-2P2-Egfp)/+^* males [originating from a line obtained by intercrossing a R*osa26^(pCAG-flSTOP-Cas9-2P2-Egfp)/+^* founder (Platt et al., 2014) to constitutive cre-expressors (Tang et al., 2002), and kept on a C57Bl/6 background] to wild-type CD1 females.

Animals were staged by timed breeding and vaginal plug inspection. When due, pregnant females were sacrificed by cervical dislocation. Animals were genotyped as follows: *Rosa26^pCAG-Cas9-2P2-Egfp)/+^* embryos were distinguished from their wild-type littermates by inspection under fluorescence microscope; *Foxg1^+/-^* embryos were distinguished from their wild-type littermates by PCR genotyping, as previously described (Muzio and Mallamaci, 2005).

Molecular sexing was performed using a dedicated procedure, run in parallel with the microdissection of neural tissue of interest. For this purpose, a skin fragment from each embryo was collected and DNA extracted from it was used for fast, PCR-based genotyping. Males were distinguished by an oligo pair specifically amplifying the Y-chromosome-located *Uty* gene (see Table S6). In general, extraction of genomic DNA employed for genotyping and preparation of the PCR reaction mix were performed by a KAPA HotStart Mouse Genotyping Kit Roche (KK7351), according to the manufacturer's instructions.

### Primary neocortical cultures: early-, mid- and late-neuronogenic

E11.5-E12.5 mouse neocortical primordia were dissected and mechanically dissociated to single cells by gentle pipetting. Dissociated cells were quantified in a Burker chamber and then plated in 24-multiwell plates (Falcon) at 100-300 cells/μl, in pro-proliferative medium [1:1 DMEM-F12, 1×Glutamax (Gibco), 1×N2 supplement (Invitrogen), 1 mg/ml BSA, 0.6% glucose, 2 μg/ml mouse heparin (StemCell Technologies), 1×penicillin/streptomycin (Gibco), 10 µg/ml fungizone (Gibco), 20 ng/ml bFGF (Invitrogen) and 20 ng/ml EGF (Invitrogen)]. If required, neural cells were transduced with a LV mix, each LV at a multiplicity of infection (m.o.i.) ≥8, sufficient to infect almost the totality of neural cells under these conditions (Brancaccio et al., 2010). Neural cells were subsequently cultured according to three different schedules, aiming to model early, mid and late phases of neuronogenic progression as follows.

(1) Protocol I (Prot-I): early-neurogenic cultures were plated in pro-proliferative medium (see above) at 300 cells/µl, kept in such medium until days 1-3 *in vitro* (DIV1-DIV3), and then processed for analysis.

(2) Protocol II (Prot-II): mid-neuronogenic cultures were plated for 20 h in 5% FBS-supplemented pro-proliferative medium (see above) at 300 cells/µl. Cells were kept in the resulting medium up to DIV3 and then processed for analysis.

(3) Protocol III (Prot-III): late-neuronogenic cultures were plated in pro-proliferative medium (see above) at a 100 cells/µl; 20 h later, their medium was further supplemented with 5% FBS; next, half of the medium was replaced daily with fresh medium 1:1 DMEM-F12 1× Glutamax (Gibco), 1× N2 supplement (Invitrogen), 1 mg/ml BSA, 0.6% glucose, 2 μg/ml mouse heparin (Stem Cell Technologies), 1× penicillin/streptomycin (Gibco), 10 µg/ml fungizone (Gibco) and 5% FBS (Gibco) up to DIV9, when cells were processed for analysis. When due, medium was further supplemented from DIV4 to DIV9 with 10 µM lamivudine (L1295-10MG, Sigma-Aldrich), assuming a conventional 3 days drug half-life.

In general, lentiviral transgenes were activated at day *in vivo* 0 (DIV0, i.e. the dissection day) using 2 µg/ml doxycycline (Sigma, D9891-10G) medium supplementation, and kept switched on by further doxycycline supplementation, performed assuming a conventional 2 days drug half-life.

### Primary neocortical cultures: neuron enriched

Cortical tissue from E16.5 mice was chopped to small pieces for 5 min, in the smallest possible volume of ice-cold 1×PBS, 0.6% D-glucose and 5 mg/ml DNaseI (Roche, 10104159001). After enzymatic digestion in 2.5×trypsin (Gibco, 15400054), 2 mg/ml DNaseI for 5 min and its inhibition with DMEM-glutaMAX (Gibco), 10% FBS (Euroclone) and 1×penicillin/streptomycin, cells were spun down and transferred to differentiative medium [Neurobasal-A, 1×Glutamax (Gibco), 1×B27 supplement (Invitrogen), 25 µM L-glutamate (Sigma), 25 µM β-mercaptoethanol (Gibco), 2% FBS, 1×penicillin/streptomycin (Gibco) and 10 µg/ml fungizone (Gibco)]. Cells were counted and plated onto 0.1 mg/ml poly-L-Lysine (Sigma, P2636) pre-treated 12-multiwell plates (Falcon) at 8×10^5^ cells per well in 0.6-0.8 ml differentiative medium. Cytosine β-D-arabinofuranoside (AraC; 10 µM, Sigma, C6645) was added to the medium at DIV1. Cells were kept in culture for 8 days.

When required, lentiviral culture transduction was performed at DIV1, and TetON-regulated transgenes were activated, generally by 2 µg/ml doxycycline (Sigma, D9891-10G) medium supplementation at DIV4. For the results shown in Fig. 10 only doxycycline was employed at 200 ng/ml, starting from DIV2.

### Lentiviral vectors

Third generation self-inactivating (SIN) lentiviral vectors (LVs) were generated as previously described (Follenzi and Naldini, 2002) with minor modifications. HEK293T cells were resuspended in DMEM glutaMAX, 10×FBS and 1×penicillin/streptomycin, and plated on 10 cm diameter plates at 8×10^6^ cells/plate. Three days later, they were co-transfected with the transfer vector plasmid plus three auxiliary plasmids (pMD2-VSV.G, pMDLg/pRRE and pRSV-REV) in the presence of LipoD293 (SigmaGen, SL100668). The conditioned medium was collected 24 and 48 h after transfection, filtered and ultracentrifuged at 50,000 ***g*** on a fixed angle rotor centrifuge (JA25.50 Beckmann Coulter) for 150 min at 4°C. Lentiviral pellets were then resuspended in (BSA-free) 1×PBS (Gibco). LVs were titrated by real-time quantitative PCR after infection of HEK293T cells, as previously reported (Sastry et al., 2002). One end-point fluorescence-titrated LV was included in each PCR titration session and PCR titers were adjusted to fluorescence-equivalent titers throughout the study.

The full list of LVs employed for this study is reported in Table S7. Performances of *Foxg1*-modulating LV transgenes were monitored by qRT-PCR. Results are summarized in Table S2.

### Fluorescence-activated cell sorting

Acute preparations of early proliferating neocortical precursors were transduced by lentiviral mixes including LV_pTα1-mCherry, which expresses the corresponding red fluoroprotein in committed neuronogenic progenitors and their postmitotic progenies. Four days later, the resulting neurospheres were treated with 1×trypsin at 37°C for 2 min, transferred to 10%FBS-containing medium for trypsin inactivation, and dissociated by gentle pipetting. Neural cells were pooled, spun at 200 ***g*** and resuspended at 1×10^6^ cells/ml in a dedicated flow cytometry buffer (a Phenol Red-free medium including 1×PBS, 25 mM HEPES and 2% FBS). Cell suspensions were filtered using a 70 µm strainer (pluriSelect, 43-10070-70), and transferred to flow cytometer tubes (pluriSelect, 05-03040-01). Cells were profiled using a Biorad S3 Cell cytofluorimeter. First, forward scatter (FSC) and side scatter (SSC) parameters were used to exclude debris and cell aggregates. Next, analytical gates R3-R4 were set for alternative mCherry^+/−^ categorizations. mCherry^−^ and mCherry^+^ preparations (highly enriched in NSCs and NPs+Ns, respectively) were collected and employed for subsequent RNA profiling.

### Genomic DNA isolation and qPCR amplicon quantitation

DNA was isolated from neocortical tissue as well as from primary pallial cultures. Neocortices from E14.5 embryos and P0 pups were microdissected, cut into small pieces for <5 min in the smallest possible volume of ice-cold 1×PBS and 0.6% glucose, and kept on ice. Minced neural tissue was further dissociated to single cells by 2×trypsin at 37°C for 5 min, followed by gentle pipetting and enzyme inactivation by FBS. On the other side, primary cell cultures were immediately treated with 0.3×trypsin at 37°C for 5 min, again followed by gentle pipetting and enzyme inactivation by FBS. Next, in both cases, cells were counted in a Burker chamber, split into aliquots of 10^6^ cells each, pelleted for 5 min at 200 ***g*** and stored at −80°C for subsequent use.

Single cell aliquots were processed by the FlexiGene DNA Kit; Qiagen, according to the manufacturer's instructions, with the following modifications: (1) PK was employed at 0.6 mg/ml (high PK) and 1.2 mg/ml (very high PK); (2) PK sample incubation was extended to 6 h; and (3) following precipitation, the DNA pellet was washed three times with 70% ethanol. Finally, DNA was resuspended in water and quantified using a DS-11 spectrophotometer (DeNovix).

Quantification of genomic amplicons (*L1* elements and X-chromosome genes) was performed starting from 10 ng DNA per each reaction, by means of the SsoAdvanced SYBR Green SupermixTplatform (Bio-Rad), according to the manufacturer's instructions. PCRs were run on a Bio-Rad CFX96TM Thermal Cycler. Primer sequences and thermal reaction profiles are reported in Table S6. Each amplicon was qPCR analyzed in technical triplicate, and results were averaged. Averages were normalized against levels of selected autosomal amplicons, as reported in the figures (depending on cases, the level of a single reference gene or the geometrical average of more than one of them were employed). As specified in the figure legends, biological replicates were DNA preparations originating (1) from different embryo/neonate individuals or (2) from individually transduced and cultured cell aliquots, taken from pooled neural cells preparations.

### Validation of family-specific diagnostic mL1 primers

Genomic DNA extracted from P0 wild-type neocortices was employed as a substrate to obtain mL1 amplicons. PCRs were primed by family-specific oligonucleotide pairs [A5utr.t3L(oM)/F and A5utr.t3L(oM)/R; Gf5utr.t3L(oM)/F and Gf5utr.t3L(oM)/R; Tf5utr.t1L(M)/F and Tf5utr.t1L(M)/R] and a 3'UTR-specific pair [AM.mL1-pan3utr/F and AM.mL1-pan3utr/R]. They were catalyzed over 40 cycles by SsoAdvanced, as described in Table S6. Amplicons were cloned using a TOPO-TA cloning kit (Invitrogen, K4575J10), according to the manufacturer's instructions. For each amplicon, at least six clones were double-strand sequenced using the Sanger method, by a Commercial operator (Eurofins). Sequences were aligned by Clustal Omega and further processed to generate experimental, SEQ.A.x, SEQ.Gf.x, SEQ.Tf.x and SEQ.3'UTR consensuses. Similarly, harvested from the Dfam database (https://www.dfam.org) under accession numbers DF0001807, DF0001809, DF0001811, DF0001816, DF0001819, DF0001821, DF0001823, DF0001849, DF0001851, DF0001864, DF0001866, DF0001868, DF0001806, DF0001808, DF0001810, DF0001815, DF0001818, DF0001820, DF0001822, DF0001848, DF0001850, DF0001863, DF0001865 and DF0001867, family specific L1 5'UTR and 3'UTR sequences were aligned by Clustal Omega and further processed to generate family-specific DFAM.A, DFAM.Gf and DFAM.Tf and pan-L1 DFAM.3′UTR consensuses. Finally, each SEQ consensus was aligned against the corresponding DFAM consensus.

### Chromatin immunoprecipitation assay

Chromatin immunoprecipitation-quantitative polymerase chain reaction assays (ChIP-qPCRs) were performed on chromatin aliquots prepared from 3.0×10^5^ cells (αFoxg1-ChIP) or 1.0×10^5^ cells (αH3K4me3-, αH3K9me3-, αH3K27ac- and αMeCP2-ChIP). ChIP analysis was performed using the MAGnify Chromatin Immunoprecipitation System kit (Invitrogen), according to the manufacturer's instructions, with minor modifications. Briefly, chromatin was fixed by 1% formaldehyde for 10 min at room temperature. After cell lysis, fixed chromatin was sonicated by a Soniprep 150 apparatus according to the following settings: (1) on ice; 5 s ON, 55 s OFF; oscillation amplitude 5 μm; 4 cycles (αFoxg1-ChIP); and (2) on ice; 5 s ON, 55 s OFF; oscillation amplitude 5 μm; 5 cycles (αH3K4me3-, αH3K9me3-, αH3K27ac- and αMeCP2-ChIP). Agarose gel electrophoresis was employed to estimate quality of sonicated chromatin.

Sonicated chromatin was immunoprecipitated for 2 h at 4°C in a final volume of 100 μl, keeping the tubes in a rotating device, using the following, agarose bead-bound antibodies: anti-Foxg1 (rabbit polyclonal, Abcam, ab18259), 10 μg/reaction; anti-H3K4me3 (rabbit polyclonal, Abcam, ab8580), 3 μg/reaction; anti-H3K9me3 (rabbit polyclonal, Active Motif, 39161), 3 μg/reaction; anti-H3K27ac (rabbit polyclonal, Abcam, ab177178), 3 μg/reaction; and anti-MecP2 (rat polyclonal IgG2a serotype, Active Motif, 61291), 3 μg/reaction. For this purpose, each antibody was pre-coupled to 10 μl of protein A/G Dynabeads (ThermoFisher, 492024).

Next, immunoprecipitated DNA was purified according to the manufacturer's instructions using the MAGnify Chromatin Immunoprecipitation System (ThermoFisher, 492024). Finally, 1/30 of each immunoprecipitated (IP) DNA sample was amplified by qPCR. For each sample, qPCRs were performed in technical triplicate. Averages were normalized against input chromatin and further normalized against controls. Experiments were performed at least in biological triplicate. Results were evaluated using an unpaired Student's *t*-test, via Excel software.

### Total RNA extraction

Total RNA was extracted from both primary neural cultures and acutely dissected neocortical samples using TRIzol Reagent (Thermofisher, 15596026) according to the manufacturer's instructions. RNA was precipitated using isopropanol and GlycoBlue (Ambion) overnight at −80°C. After two washes with 75% ethanol, RNA was resuspended in 20 µl sterile nuclease-free deionized water. Agarose gel electrophoresis and spectro-photometric measurements (DS-11, DeNovix) were employed to estimate its concentration, quality and purity.

### RNA-immunoprecipitation

RNA immunoprecipitation (RIP) was performed starting from primary neural cultures. Before starting cell processing, for each RIP reaction, 10 µl of protein A/G Dynabeads (Thermofisher, 492024) were coupled with 10 µg of αFoxg1 (ChIP-grade, rabbit polyclonal, Abcam, ab18259) or 10 µg of rabbit IgG (Millipore, 12370) as control, according to the manufacturer's protocols. Pre-clearing control beads were prepared omitting antibody coupling.

Cells were washed once with ice-cold 1×PBS. 75 µl ice-cold lysis buffer was added to each well (of a 12-multiwell plate) and kept on ice for 10 min. Next, cells were scraped and lysed by vigorously pipetting up and down, paying attention not to make bubbles. Lysate collected from 10 wells (about 8×10^6^ cells; corresponding to a αFoxg1/IgG biological samples pair) was pipetted up and down and kept for 10 min on ice twice, then it was centrifuged at 2000 ***g*** for 10 min at 4°C, and then centrifuged at 16,000 ***g*** for 10 min at 4°C. The supernatant resulting from each sample was incubated with pre-clearing beads (pre-equilibrated in lysis buffer) for 30 min at 4°C on roller-shaker. Preclearing beads were then separated with a magnet, and supernatant was incubated with antibody-coupled beads (pre-equilibrated in lysis buffer) overnight at 4°C on roller-shaker. 10% of supernatant (Input, IN-RIP) was stored on ice. The day after, beads were collected with a magnet and washed five times with 0.5 ml ice-cold high-salt buffer [lysis buffer: 25 mM Tris-HCl, 150 mM KCl (Ambion), 10 mM MgCl_2_ (Ambion), 1% (vol/vol) NP-40 (Thermo Fisher Scientific), 1×EDTA-free protease inhibitors (Roche), 0.5 mM DTT (Invitrogen), 10 µl/ml rRNasin (Promega) and 10 µl/ml SuperaseIn (Applied Biosystems); high-salt buffer: 25 mM Tris-HCl, 350 mM KCl (Ambion), 10 mM MgCl_2_ (Ambion), 1% (vol/vol) NP-40 (Thermo Fisher Scientific), 1×EDTA-free protease inhibitors (Roche) and 0.5 mM DTT (Invitrogen)]. For each sample, RN- immunoprecipitated (IP-RIP) and input were extracted with Trizol LS reagent according to the manufacturer's instructions. For each sample, a supplementary extraction was used to improve the total RNA yield. RNA was precipitated using isopropanol and GlycoBlue overnight at −80°C. After two washes with 75% ethanol, the RNA was resuspended in 10 µl sterile nuclease-free deionized water. Agarose gel electrophoresis and spectrophotometric measurements (NanoDrop ND-1000) were employed to estimate quantity, quality and purity of the resulting RNA.

### Total and immunoprecipitated RNA quantitation

RNA preparations from total RNA samples, and RIP samples were treated by TURBO DNase (2 U/µl) (Thermofisher, AM2238) for 1 h at 37°C, following the manufacturer's instructions. cDNA was produced by reverse transcription (RT) by Superscript III (ThermoFisher, 18080093) according to the manufacturer's instructions, in the presence of random hexamers. The RT reaction was diluted 1:5 (in case of both RIP and total RNA samples), and 1 µl of the resulting cDNA was used as substrate of any subsequent quantitative PCR (qPCR) reaction. Limited to intron-less amplicons and for RIP-derived samples, negative control PCRs were run on RT(−) RNA preparations. qPCR reactions were performed on the SsoAdvanced SYBR Green Supermix platform (Bio-Rad, 1725270), according to the manufacturer's instructions. For each transcript under examination and each sample, cDNA was qPCR analyzed in technical triplicate, and results averaged. In the case of total RNA, mRNA levels were normalized against the geometrical average of *Rpl10a*,*Gapdh*, *Cltc* and *Rn18S* levels (or a subset of them, see legends to figures). In the case of RIP samples, IP samples were normalized against inputs. Primer sequences are reported in Table S6. Data analysis was performed using Microsoft Excel.

### Immunofluorescence: sample preparation

Brains were dissected out from P0 mouse pups, fixed in 4% paraformaldehyde at 4°C overnight, transferred to 30% sucrose and 1×PBS, kept at 4°C until equilibration, incubated in OCT (Bio-Optica) and sliced at 30 µm. Slices were attached to Superfrost N^+^ glass slides (ThermoFisher Scientific), which were stored at −80°C. Before immunolabeling, slides were transferred to room temperature for 10 min and washed in 1×PBS for 30 min for OCT removal.

In case of primary neural cell preparations, cells were dissociated by 0.3×trypsin digestion for 4 min at room temperature, followed by gentle (10 times) pipetting and 1:1 v/v trypsin inactivation by FBS-containing medium. Cells were resuspended at 200 cells/μl and 1 ml of suspension was plated on a 12 mm diameter glass coverslip previously coated with 0.1 mg/ml poly-L-lysine. Cells were kept in 5% CO_2_ at 37°C for 1 h, fixed in 4% PFA at room temperature for 15 min, and finally washed three times in 1×PBS.

Fixed and/or washed cells and/or brain sections were treated with blocking mix (1×PBS, 10% FBS, 1 mg/ml BSA and 0.1% Triton X-100) for at least 1 h at room temperature. Next, they were incubated with primary antibodies in blocking mix, overnight at 4°C. The day after, samples were washed three times in 1×PBS, 0.1% Triton X-100 (5 min each) and then incubated with secondary antibodies in blocking mix, for 2 h at room temperature. Samples were finally washed three times in 1×PBS, 0.1% Triton X-100 (5 min each), subsequently counterstained with DAPI (4′, 6′-diamidino-2-phenylindole) and mounted in Vectashield Mounting Medium (Vector). The following primary antibodies were used: anti-Sox2, rabbit polyclonal (clone 2Q178, Abcam, ab6142, 1:400); anti-Tubb3, mouse monoclonal (clone Tuj1, Covance, MMS-435P, 1:1000); anti-Foxg1 antibody (rabbit polyclonal, Abcam, ab18259, 1:500); anti-RFP (specifically recognizing mCherry), rat monoclonal (Antibodies online, ABIN334653, 1:500); anti-EGFP (Enhanced Green Fluorescent Protein) and chicken polyclonal (GenTex, GTX13970, 1:1000). Secondary antibodies were conjugates with Alexa Fluor 488, Alexa Fluor 594 or Alexa Fluor 647 fluorophores (Invitrogen, 1:500).

### Image acquisition and analysis

Immunostained cells and/or brain sections were photographed on a Nikon Eclipse TI microscope, equipped with a 20× objective, using a Hamamatsu 1394 ORCA-285 camera (Fig. 2) or a Nikon C1 confocal system (Fig. 5, Fig. S2). Hamamatsu photos were collected as 1344×1024 pixel files. Nikon C1 photos were collected as 3 µm *z*-stacks (0.3 µm steps) of 1024×1024 pixel images. Images were analyzed using Adobe Photoshop CS6 (Fig. 2) and Volocity 5.5.1 (Fig. 5, Fig. S2) software. Resulting numerical data were further processed using Microsoft Excel software.

Specifically in case of the analysis shown in Fig. 5, the following strategy was implemented. After acute transduction with LV_pTα1-mCherry (firing in NPS and Ns) at moi=8, NSCs, NPs and Ns were recognized by their mCherry^−^Tubb3^−^, mCherry^+^Tubb3^−^ and mCherry^±^Tubb3^+^ profiles, respectively. Moreover, for each cell, nuclear Foxg1 protein content was quantified by Volocity 5.5.1 analysis of aFoxg1-IF signal. Data referring to an equal number of cells from six biological replicates of control samples were collected, cumulatively ranked and employed to establish boundaries between contiguous (aFoxg1-IF signal) deciles (here, biological replicates are aliquots of neural cells originating from the same starting pool, each independently transduced and cultured). Next, starting from six and five biological replicates of control and *Foxg1*-OE samples, respectively, distinctive cell types (NSCs, NP and Ns) of different genotypes (control or mis-expressing Foxg1), falling within different decile bins were quantified, normalized against total cells of the same type and genotype, and finally plotted against decile number. Cumulatively, over 17,000 neural cells were scored for this analysis.

As for Fig. S2, Foxg1 protein level were revealed by anti-Foxg1/anti-rabbit-Alexa488 immunofluorescence, and quantified by Volocity 5.5.1 analysis, run over the entire cell population. For each test, at least six biological replicates were employed. Here, biological replicates are columns of midparietal neocortex taken from different brains, each including at least 2500 cells (Fig. S2A), or aliquots of neural cells originating from the same starting pool, each independently transduced and cultured (Fig. S2B,C).

### Statistical analysis

When not otherwise stated, experiments were performed at least in biological triplicate. Statistical tests employed for result evaluation, *P*-values and definitions of *n* (number of biological replicates) are provided in each figure. Differences were considered statistically significant if *P*<0.05. In all figures the notation ‘*n*=*a,b,...,k*' is used to indicate that *n* equals *a*, *b*...and *k*, in cases where samples share the different α, β....and κ states of the independent variable, respectively. Full primary data referred to in all figures are reported in Table S5.

## Supplementary Material



10.1242/develop.202292_sup1 Supplementary information

Table S5. Full primary data (referring to: Figures 1,2,3,4,5,6,8,9,10,11,12,13,14,15,16,S2,S3,S4,S5)
