# Supplementary material for: Foxg1 bimodally tunes L1-mRNA and -DNA dynamics in the developing murine neocortex
Source: Development. 2024 May 23;151(10):dev202292. doi: 10.1242/dev.202292 (PMC11190451; doi:10.1242/dev.202292)
Supplement: Supplementary information [file develop-151-202292-s1.pdf]

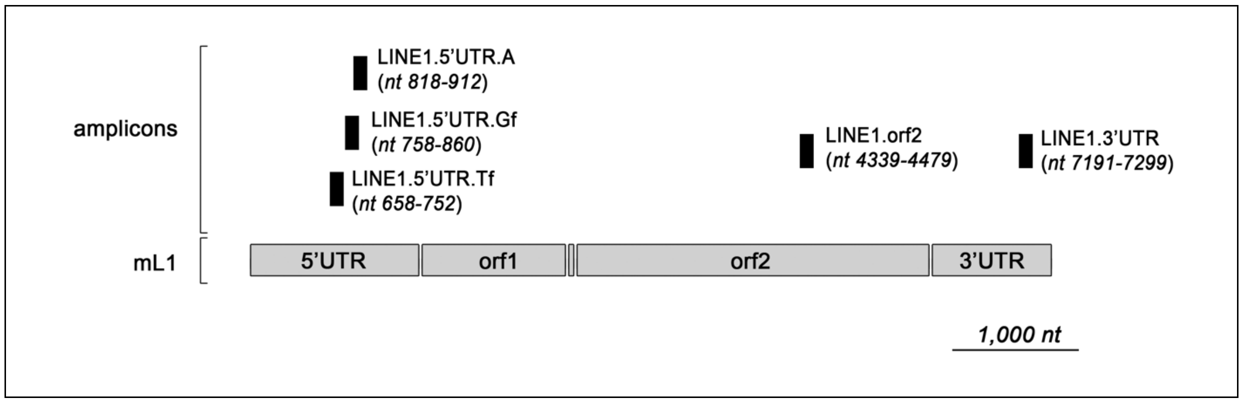

Fig. S1. Mapping diagnostic amplicons to mLINE1 elements. Shown are the five amplicons employed in this study and their locations. Coordinates refer to an idealized mLINE1 element obtained by juxtaposing: (a) Clustal Omega alignement of subfamily specific 5'UTRs (Dfam DF0001807, DF0001809, DF0001811, DF0001816, DF0001819, DF0001821, DF0001823, DF0001849, DF0001851, DF0001864, DF0001866 and DF0001868), (b) Clustal alignement of subfamily specific orf1 & orf2 elements reported in "Sookdeo et al (2013), doi: 10.1186/1759-8753-4-3, Additional File 1", and (c) Clustal Omega alignement of subfamily specific 3'UTRs (Dfam DF0001806, DF0001808, DF0001810, DF0001815, DF0001818, DF0001820, DF0001822, DF0001848, DF0001850, DF0001863, DF0001865, and DF0001867).

Development • Supplementary information

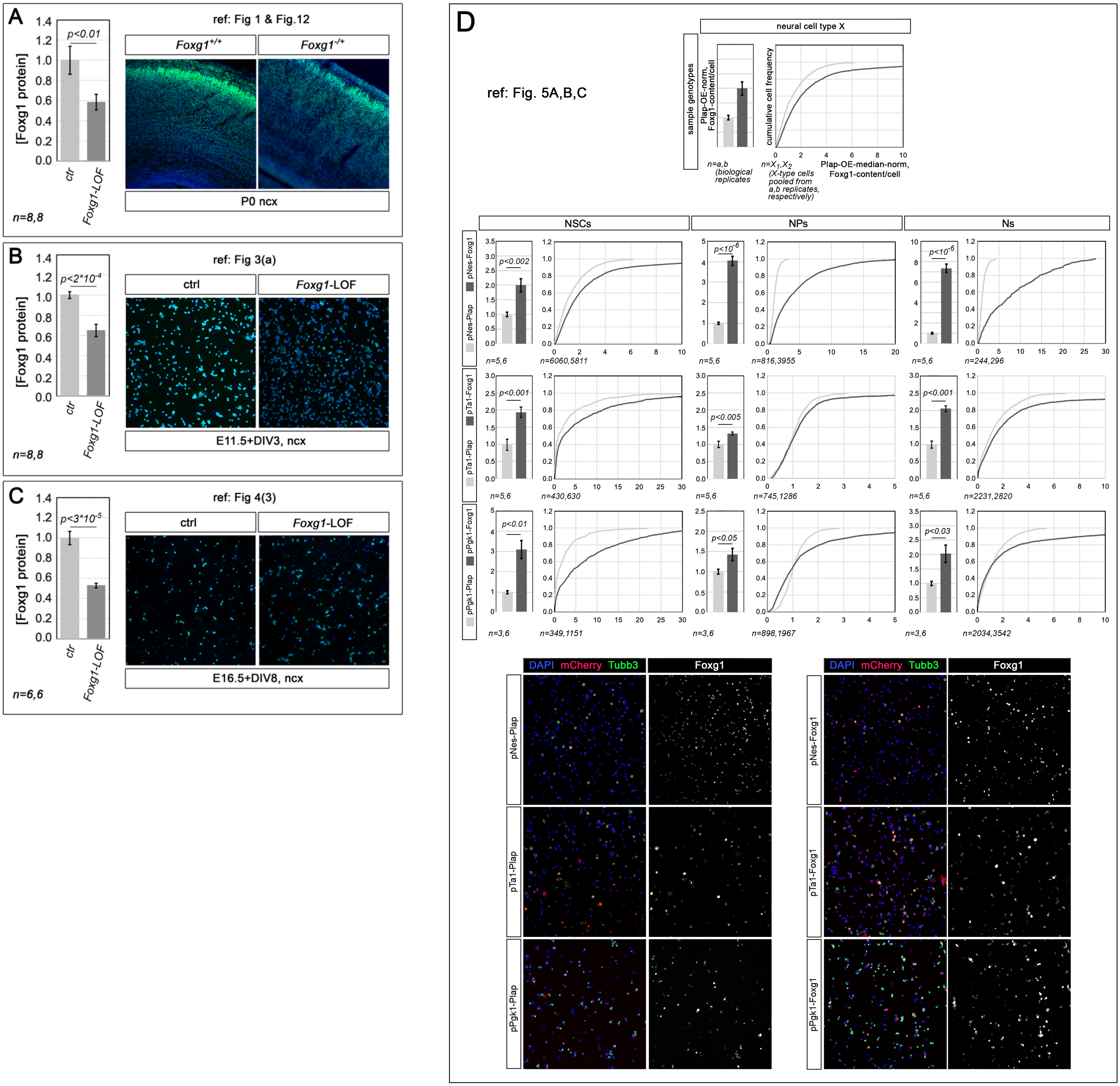

Development • Supplementary information

Fig. S2. Foxg1 protein dynamics upon selected LOF- and GOF- manipulations. Here, reported are control-normalized Foxg1 protein levels in selected experiments of this study, each with reference Figures and panels, and indicative examples of the corresponding immunofluorescences. To note, Foxg1 protein downregulation was achieved by heterozygosity for a *Foxg1*-null allele (**A**), Cas9/sgRNA-driven knock-down (**B**) and shRNA-driven interference (**C**), Foxg1 protein upregulation by Tet^{ON}-controlled expression of a Foxg1-encoding transgene, driven by pNes, pTa1 and pPgk1 promoters (**D**). In case of overexpression assays, in addition to quantifications of average expression levels peculiar to distinctive neural cell types (NSCs, NPs, and Ns), shown also are cumulative distributions of protein levels displayed by single cells of different neural types. In such case, for the sake of clarity, a graph prototype with parameters represented in *x* and *y* is reported at the top of the panel. Error-bars representing sem's. Statistical significance of results evaluated by t-test (one-tailed, unpaired). *n* generally is the number of biological replicates, i.e. mouse individuals (A) and independently cultured and engineered cell aliquots, originating from pooled, *wild-type* E16.5 (B,C) or E11.5 (D) neocortical primordia. In the only case of graphs representing cumulative distributions (D), *n* is the number of cells pooled from distinctive biological replicates of each sample.

Development • Supplementary information

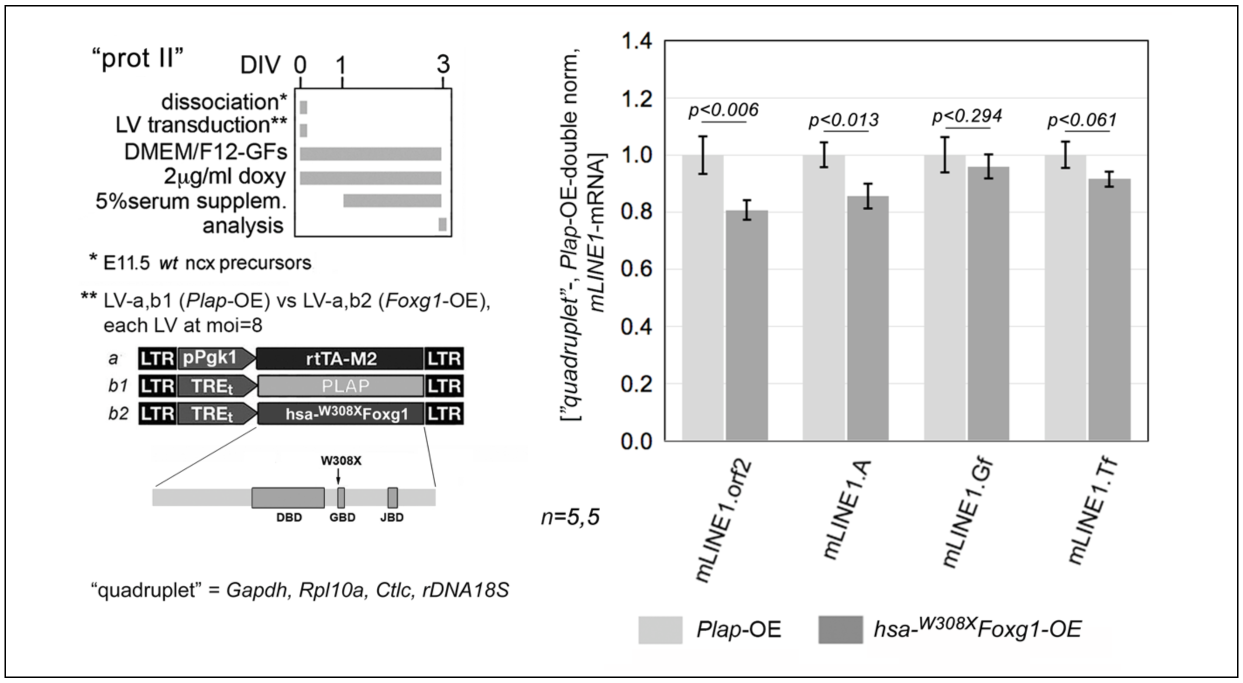

Fig. S3. Modulation of *L1*-mRNA levels in murine mid-neuronogenic pallial cultures overexpressing the *hsa-^{W308X}Foxg1* mutant allele. To left, protocol and lentiviral vectors employed. Transgenes driven by the constitutively firing pPgk1 promoter. The human (hsa) mutant allele in order encodes for a prematurely truncated protein, including the DNA-binding domain (DBD), but not the Groucho- and Jarid-binding domains (GBD and JBD, respectively). RT-PCR quantitation of pan-*L1* diagnostic amplicon "*L1*.orf2", and family-specific amplicons, "*L1*.A", "*L1*.Gf" and " *L1*.Tf", in neural cultures set according to a type II protocol. Data double normalized against gene quadruplet (*Gapdh*, *Rpl10a*, *Cltc* and *rDNA 18S*) and control values. Error-bars representing sem's. Statistical significance of results evaluated by t-test (one-tailed, unpaired). *n* is the number of biological replicates, i.e. independently cultured and engineered cell aliquots, originating from pooled, *wild-type* E11.5 neocortical primordia.

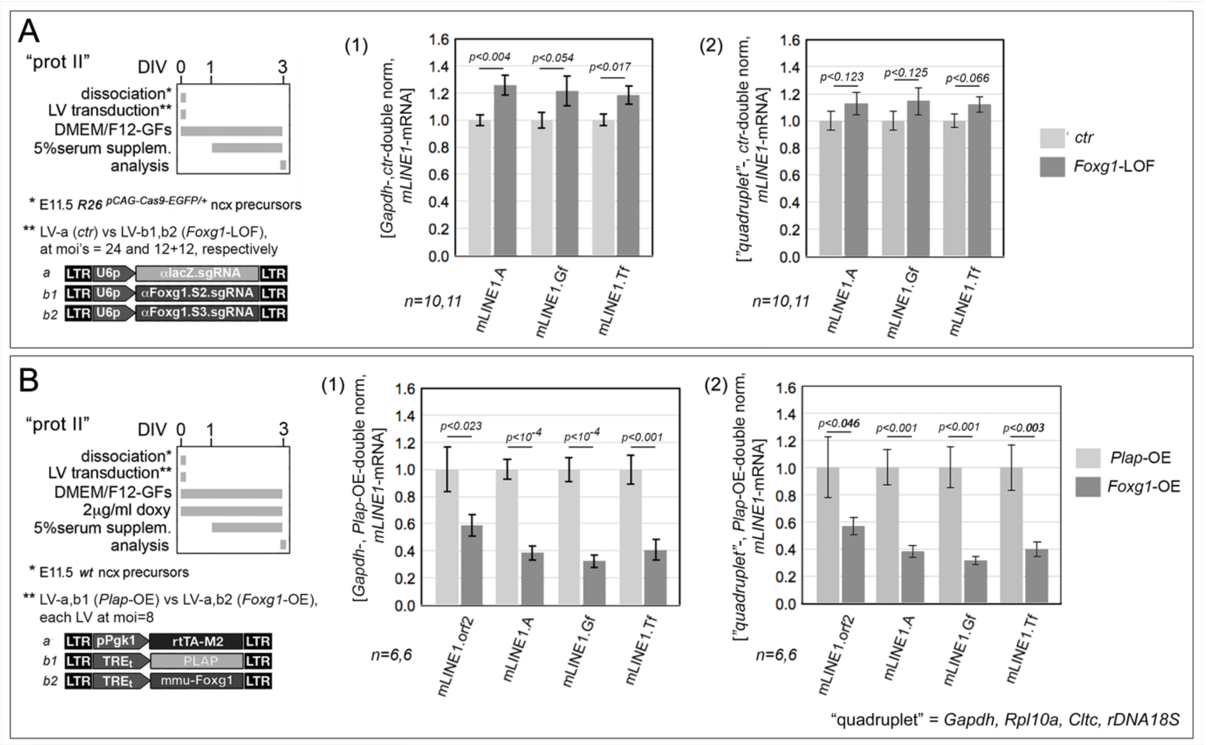

Fig. S4. Comparative evaluation of "quadruplet-" vs *Gapdh*-normalization of *L1*-mRNA expression levels. In (A) and (B) shown are data referred to in Fig. 3A and 3D, respectively, upon alternative normalization against *Gapdh* (1) and gene "quadruplet" (2).

Development • Supplementary information

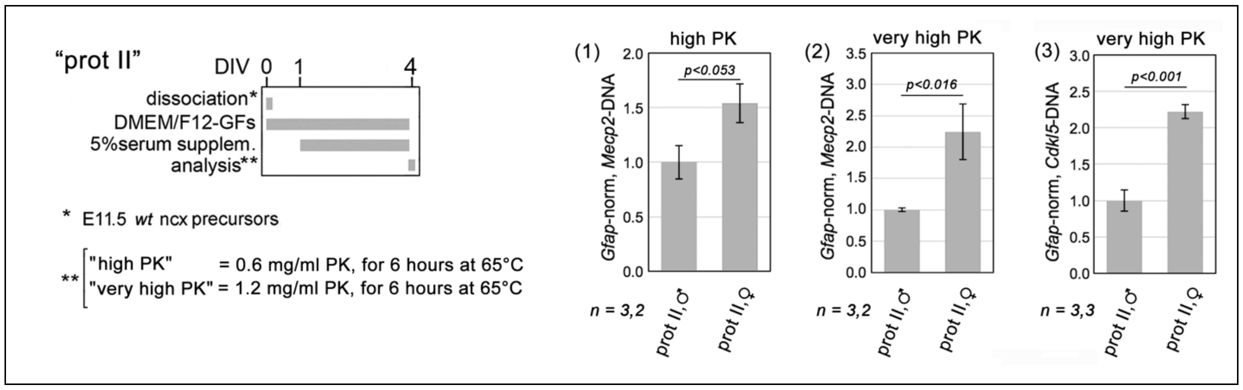

Fig. S5. Optimization of genomic DNA extraction. To left, protocol. Mid- neuronogenic cultures set by means of type II protocols. DNA extraction performed by a strong ("high PK", see Materials and Methods) or a very strong procedure ("very high PK", see Materials and Methods). To right, results. Graphs (1-3) providing a comparative assessment of "high PK" and "very high PK" procedures for their capability to extract with similar efficacy genomic DNA, regardless of chromatin accessibility. Such assesment was done by quantifying X-chromosomal *Mecp2* and *Cdkl5* sequences, amplified from female and male genomic DNA, and normalizing them against the euchromatic *Gfap* autosomal locus. Error-bars representing sem's. Statistical significance of results evaluated by t-test (one-tailed, unpaired). *n* is the number of biological replicates, i.e. independently cultured and engineered cell aliquots, originating from pooled, *wild-type* E11.5 neocortical primordia.

Development • Supplementary information

Table S1. Validating LINE1-diagnostic PCR primers. Sequences of DNA strings amplified by distinctive, family-specific (5'UTR) and shared (3'UTR), LINE1 primers were aligned by Clustal Omega and further processed to generate experimental, SEQ.A.x, SEQ.Gf.x, SEQ.Tf.x and SEQ.3'UTR.x consensuses. Similarly, subfamily specific LINE1 5'UTR and 3'UTR sequences harvested from Dfam database (listed in Legend to Fig. S1) were aligned by Clustal Omega and further processed to generate family-specific DFAM.A, DFAM.Gf and DFAM.Tf consensuses, as well as a shared DFAM.3'UTR consensus. Finally, each SEQ consensus was aligned against the corresponding DFAM consensus. Throughout the table, nucleotide numbers refer to the outputs of Clustal Omega alignments among Dfam consensus sequences.

[1] *A family-specific primers: A5utr.t3L(oM)/F, A5utr.t3L(oM)/R*

```
SEQ.A.x           GAATACTCTGCCCACTGAAACTAAGGA--GAGTGCTACCCT-CCAGRTCTGCTYATAGAGGCTAACAGAGTCACCKGAAGAACAAGCTCTTAAC
DFAM.A       818  GAATACTCTGCCCACTGAAACTAAGGA--GAGTGCTACCCT-CCAGGTCTGCTBATAGAGGCTAACAGAGTCACCTGAAGAACAAGCTCTTAAC  912
                  *********************************************************************************************
DFAM.Gf      818  GACTGCTCTGACCACTGAAACTCAGAGAAGAGAGCTTGTCTCCCACGCCTGCTGATAGAGGGTAACAAAATCAACAGAGGAACAATCTCTTAAC  912
                  ** * ***** *********** **     *** ***    ** *** * ****************** * *** * ** ****** *******
DFAM.Tf      818  GAGTGCTCTGAGCACTGAAACTCAGAGGAGAGAATCTGTCTCCCAGGTCTGCTGATAGACGGTAACAGAATCACCAGAAGAACAATCTCTAAAC  912
                  ** * *****  ********** **     *** *      ** **************** ********* ***** ********* *******
NB. "-" replaced by "C" in LINE1.A.III-VII
```

[2] *Gf family-specific primers: Gf5utr.t3L(oM)/F, Gf5utr.t3L(oM)/R*

```
SEQ.Gf.x          GGAGGTCCAAACACCAGATAACTGTACACCTTCCCTGAGAGAGGAGAGCTTGCCTACAGAGACTGCTCTGACCACTGAAACTCAGAGAAGAGAGCTTGTCTCCCACGCCTGCT
DFAM.A       758  KCTGGTTCGAACACCAGATATCTGGGTACCTGCCTTGCAAGAAGAGAGCTTGCCTGCAGAGARTACTCTGCCCACTGAAACTMAGGAKAGTGCTASYCTC--CCAGGTCTGCT  870
                  *  *** * *********** ***   **** ***** **** ****************** * ***** *************   ** *    * **  *** * *****
DFAM.Gf      758  GGAGGTCCAAACACCAGATAACTGTRCACCTTCCCTGARAGAGGAGAGCTTGCCTACAGAGACTGCTCTGACCACTGAAACTCAGAGAAGAGAGCTNGTCTCCCACGCCTGCT  870
                  ****************************************************************************************************************
DFAM.Tf      758  GGAGGTCCAAAWACAAGATATCTGCGCACCTTC---CCTGTAAGAGAGCTTGCCAGCAGAGAGTGCTCTGAGCACTGAAACTCAGAGGAGAGAATCTGTCTCCCAGGTCTGCT  870
                  *********** ** ***** *** * **** *        * ***********  ****** ******** *************** *****   ********* * *****
NB. "T" replaced by "-" in LINE1.Gf.II
```

[3] *Tf family-specific primers: Tf5utr.t1L(M)/F, Tf5utr.t1L(M)/R*

```
SEQ.Tf.x.         GAGAGTCTGTAC------------------CACCTGGGAACTGCCAAAGCAACACAGTGTCTGAGAAAGGTCCTGTTTTGGGCCTTCTTCTTCGG
DFAM.A       658  GAGYKKSSSVGSKGAGTCKCCBGACACCCNCAAGGDNCCWCACRGGAYYCYNCACGNGATCCTAAGACCYYYDGTGAGTGGAWCACAACTTCTGC  752
                  *** *** *  *                  **   **  **     *  * **** *  **  *  *   **      ***  *      *  *
DFAM.Gf      658  CCA-MYWKMTTCKRSKMCWGMS---MASYNGSCMSCYTNCNGGCCAAAGCAACACAGCTTCTGGGAAAGATCCTGTTTTGGGCCTTCACCTTCRG  752
                         * * *                  **    * * ******************** ***** ****************  ******
DFAM.Tf      658  GAGAGKSTGTAC------------------CWYCTGGGAACTGCCAAAGCAACACAGYKTCTGAGARAGGYCCTGTTTTGGGCCTTCTTCTTCGR  752
                  ************                  *****************************************************************
NB. "------------------" replaced by "ACAGAAGCT---GACAGC" in DFAM.Tf.III
```

[4] *3'UTR primers: AM.mL1-pan3utr/F, AM.mL1-pan3utr/R*

```
SEQ.3'UTR.x       GGTGGAACAACATTATGAACTAACCAGTACCCCDGAGBTCKTGWCTCTWGCTGCATRTGYATCARAAGATGRMCTARTHGGYCATCACTGGAAAGAGAGGCCCATTGG
DFAM.3'UTR        GGYGSAWCAACATTATGARCTAACCAGTACCCCKGAGCTCTTGACTCTAGCTGCATATRYATCAAAAGATGGCCTAGTCGGCCATCACTGGAAAGAGAGGCCCATTGG    108
                  **************************************************************************************************************
```

Table S2. Foxg1-mRNA dynamics upon OE- and LOF-manipulations of this study. Here, reported are control-normalized *Foxg1*-mRNA levels in selected experiments of this study, each with reference Figures and panels, and normalizer gene(s) employed for their evaluation.

ref		*Foxg1-mRNA manipulation*	
Fig. #	*panel, graph*	*ctr-norm Foxg1-mRNA levels*	*normalizer(s)*
1	---	0.64	*Gapdh*
3	A	0.64	*gene quadruplet**
	B	2.74	*gene quadruplet**
	C	3.63	*gene quadruplet**
	D	2.84	*gene quadruplet**
	E	4.02	*gene quadruplet**
4	graph (1)	4.84	*Gapdh*
	graph (2)	4.27	*Rpl10a*
	graph (3)	0.28	*Rpl10a*
8	NSCs	0.59	*Gapdh*
	NPs + Ns	0.40	
13	---	0.64	*Gapdh*
14	B, graph (1)	0.35	*Gapdh*
	B, graph (3)	3.05	*Gapdh*
S3	---	14.08	*"quadruplet"*

**(Gapdh; Rpl10a; Cltc and rDNA 18S)*

Table S3. "Quadruplet", RNA-pol II-transcribed normalizers: fluctuations of their mRNA levels throughout normal neuronogenesis as well as upon *Foxg1* manipulation in differentiating neurons. (A) Violin plots representing single cell mRNA levels, in pallial apical precursors (AP), basal precursors (BP), early neurons (EN) and late neurons (LN). Downloaded from http://genebrowser.unige.ch/science 2016 (Telley et al., 2016). **(B)** Modulation of average mRNA levels in differentiating neuronal cultures overexpressing *Foxg1* (Artimagnella and Mallamaci, 2020 i.e doi:10.5281/zenodo.3739467).

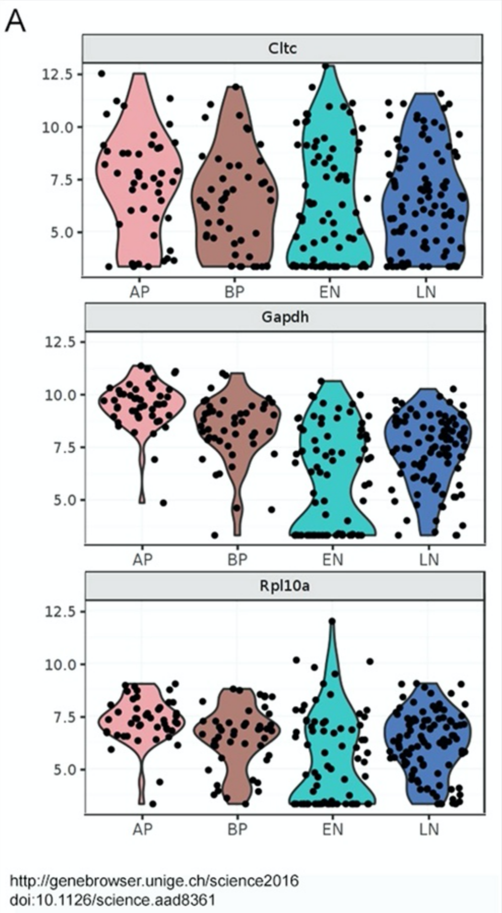

B

Locus	log2FC	logCPM	P-value	FDR	Gene Name
ENSMUSG00000047126	0,01	9,73	0,94	0,96	Cltc

Locus	log2FC	logCPM	P-value	FDR	Gene Name
ENSMUSG00000057666	-0,16	9,44	0,15	0,24	Gapdh

Locus	log2FC	logCPM	P-value	FDR	Gene Name
ENSMUSG00000037805	0,07	6,50	0,68	0,77	Rpl10a

doi:10.5281/zenodo.3739467

Development • Supplementary information

Table S4. Putative mediators of Foxg1 impact on L1 retro-transcription. Shown are selected genes impacting on L1 retro-transcription/transposition rates, (A) whose mRNA levels are sensitive to Foxg1 overexpression, or (B) whose protein products physically interact with Foxg1 protein.

Foxg1-responsive gene (ref 1)	*impact on retro-transcription/transposition*	*mRNA dynamics upon Foxg1 overexpression in ncx neurons*				
		Locus	*log2FC*	*logCPM*	*P-value*	*FDR*
Apobec1	limits Line1 retrotransposition (ref 2)	ENSMUSG00000040613	-1.01	2.02	0.00	0.00

Foxg1 interactor (ref 3)	*impact on retro-transcription/transposition*	*Foxg1-protein interaction details (Biogrid #)*
MOV10	limits Line1 retrotransposition (ref 4)	1507467
DDX39A	limits Line1 retrotransposition (ref 4)	1507432

REFERENCES

1 doi: 10.5281/zenodo.2849656.
2 doi: 10.1093/nar/gkr124
3 https://thebiogrid.org/
4 doi: 10.1371/journal.pgen.1002941

Table S5. Full primary data
(referring to: Figures 1,2,3,4,5,6,8,9,10,11,12,13,14,15,16,S2,S3,S4,S5)

Available for download at
https://journals.biologists.com/dev/article-lookup/doi/10.1242/dev.202292#supplementary-data

Development • Supplementary information

Table S6. Primer sequences and mL1 thermal reaction profiles

The following oligonucleotide pairs have been employed in this study:

Rpl10a/F,R
5′ CAG CAG CAC TGT GAT GAA GCC AAG G 3′
5′ GGG ATCT GCT TAA TCA GAG ACT CAG AGG 3′
mGapdh/F,R
5′ ATC TTC TTG TGC AGT GCC AGC CTC GTC 3′
5′ GAA CAT GTA GAC CAT GTA GTT GAG GTC AAT GAA GG 3′
mmuCltc/F,R
5′ CTT GCT CAG CGT TTG GAA AAA CAC GAA CTC AT 3'
5′ GCC AAT TCT GTG TCT TTA GAT TCA GAG GCA TAC 3'
mmuRn18s/F,R
5′ AAT TGA CGG AAG GGC ACC ACC AGG AGT 3'
5′ GCC ATG CAC CAC CAC CCA CGG AAT C 3'
Foxg1(cds)/F,R
5′ GAC AAG AAG AAC GGC AAG TAC GAG AAG C 3′
5′ GAA CTC ATA GAT GCC ATT GAG CGT CAG G 3′
Foxg1(Mid)/F,R
5′ GAC AAG AAG AAC GGC AAG TAC GAG AAG C 3′
5′ GAA CTC ATA GAT GCC ATT GAG CGT CAG G 3′
Couptf1_Foxg1-BS.a/F,R
5′ CCA TCT GAA TGC AAA ACG TAA GCA TG 3′
5′ ATG GAC CTC GCT CTA CTA ATT TGT TGG 3′
Gfap_Foxg1-BS.b/F,R
5′ GCA GGA ACT CCT GAG GCT GGC AGC ATT 3′
5 GTG TAA ACA AAA TTA CAG GAG CTT GTG TTG AAC TTG C 3'
Nfia_Foxg1-BS.h1/F,R
5′ CAG AAC TTG GTG GAT GGT GAG TTG GCA G 3′
5′ TGT GAG TTT GAT GAA TGT GAG GTC TGG GAC 3′
S100b_Foxg1-BS.x2/F,R
5′ TTG TGA GCC ACC CAA TGT GGT TCT GG 3′
5′ CCA CAA ATG TGT TGG AAA GAT ATA ACT CAA AG 3′
Sox9_Foxg1-BS.h1/F,R
5′ CCG TGA TTG GCC CGA GGT ATC TAA CGT G 3′
5′ AGT TGT CGC TCC CAC AGA AGT TTC CAG G 3′
A5utr.t3L(oM)/F,R
5′ GAA TAC TCT GCC CAC TGA AAC TAA GGA GA 3′
5′ GTT AAG AGC TTG TTC TTC AGG TGA CTC TGT 3′
Gf5utr.t3L(oM)/F,R
5′ GGA GGT CCA AAC ACC AGA TAA CTG TAC ACC 3′
5′ AGC AGG CGT GGG AGA CAA GCT CTC TT 3′
Tf5utr.t1L(M)/F,R
5′ GAG AGT CTG TAC CAC CTG GGA ACT GC 3′
5′ CCG AAG AAG AAG GCC CAA AAC AGG AC 3′
Orf2/F,R *
5′ AGC TTT YAT YCC AGG GAT GCA GGG AT 3′
5′ TGG GTG TTG GAT MTT GTC AAA TGC TT 3′
* Y=C or T; M=A or C
AM.mL1-pan3utr/F,R
5′ GGT GGA ACA ACA TTA TGA ACT AAC CAG TAC C 3′
5′ CCA ATG GGC CTC TCT TTC CAG TGA TG 3′
Cdkl5/F,R
5′ GCA CTA CTT GAA AAT AAA GGA CGA AGG ATG GC 3′
5′ CAG GCT GCG AGA TGA ACG TGA ATA TGA 3′
MeCP2/F,R
5′ GTC CCC TGC CCA CAC TAA GTA TGA CAT 3′
5′ GGG TCA CAT TGG GTA GCT GAA GGA GAT 3′
Uty/F,R
5′ TGC TAT ACT GAT CTC ACA AGG AAC AAA GAG CA 3′

Development • Supplementary information

The following oligonucleotide pairs have been employed in this study:
5′ GGA GCA CCG ATT CTG GGA ACT TAA CAG 3′

NB. mL1 amplicons were quantified by PCR, run on a BioRad CFX96 thermocycler, according to the following thermal reaction profiles:

- *5'UTRs*

1x(98°C/4 min); 40x(98°C/10 *s*, 68.4°C/15 *s*, 72°C/10 *s*, 74°C/3 *s*); 1x(72°C/10 min)

- *orf2 & 3'UTR*

1x(98°C/4 min); 40x(98°C/10 *s*, 68.4°C/15 *s*, 72°C/20 *s*, 74°C/3 *s*); 1x(72°C/10 min)

Development • Supplementary information

Table S7. Lentiviruses list

The lentiviruses (LVs) were used for this study were named according to the standard nomenclature: LV: pX-GOI, where pX is the promoter and GOI is the gene of interest. They were:
LV_pPgk1-rtTA2S-M2 (Spigoni et al., 2010)
LV_pNes-rtTA-M2, aka pNes/hsp68-rtTA2S-M2 (Brancaccio et al., 2010)
LV_pTα1-rtTA2S-M2 (Brancaccio et al., 2010)
LV_pSyn-rtTA2S-M2 (Tigani et al., 2020)
LV_TREt-PLAP (Falcone et al., 2019)
LV_TREt Foxg1 (Raciti et al., 2013)
LV_pU6 shFoxg1 (Sigma TRCN0000081746, in pLKO.1)
LV_pU6 shCtrl (Chiola et al., 2019)
LV_pTα1-mCherry (Brancaccio et al., 2010)
LV_pU6 shMov10 (Sigma TRCN0000097832, in pLKO.1)
LV_pU6 shDdx39a (Sigma TRCN0000071093, in pLKO.1)
LV_U6p-αLACZ.sgRNA [obtained by cloning the TGCGA ATACG CCCAC GCGAT guide (Platt et al., 2014) into BsmBI-cut, lentiCRISPR_v2ΔEB; lentiCRISPR_v2ΔEB was previously obtained starting from lentiCRISPR_v2 (Addgene #52961), deleting the EF1a-prom-Cas9 fragment by EcoRI/BamHI digestion, filling-in and religating]
LV_U6p-α Foxg1. S2.sgRNA sgRNA [obtained by cloning the GCCCG TCGGG CCGGA CGAGA guide (Mall et al., 2017) into BsmBI-cut, lentiCRISPR_v2ΔEB]
LV_U6p-αFoxg1. S3. sgRNA [obtained by cloning the CCCCG ACGCC TGGGT GATGC guide (Mall et al., 2017) into BsmBI-cut, lentiCRISPR_v2ΔEB]
LV_TREt-hsaFOXG1wt [built by transferring the AgeI-SalI fragment from pUC57 hsaF1WT (provided us on a commercial basis by Gene Universal) into the AgeI-SalI digested LV_TREt-IRES2- EGFP (Falcone et al., 2015)].
LV_TREt-hsaFoxg1(W308X) [built by transferring the AgeI-XhoI fragment from pUC57 hsaF1-W308X (provided us on a commercial basis by Gene Universal) into the AgeI-SalI digested LV_TREt-IRES2-EGFP (Falcone et al., 2015)].

NB. To build the last six LVs mentioned above, DNA manipulations (extraction, purification and ligation), bacterial cultures and transformations were performed according to standard methods. Restriction and modification enzymes were obtained from New England Biolabs and Promega; DNA fragments were purified from agarose gel by QIAquick Gel Extraction Kit (Qiagen); plasmid preparations were performed through DNA Plasmid Purification Kit (Qiagen). Plasmids were grown in E. Coli, Xl1-blue or ElectroMAX™ Stbl4™ Competent Cells (Invitrogen).

References

Falcone, C., Filippis, C., Granzotto, M. and Mallamaci, A. (2015). Emx2 expression levels in NSCs modulate astrogenesis rates by regulating EgfR and Fgf9. *Glia* 63, 412-422. doi:10.1002/glia.22761

Mall, E. M., Herrmann, D. and Niemann, H. (2017). Murine pluripotent stem cells with a homozygous knockout of Foxg1 show reduced differentiation towards cortical progenitors in vitro. *Stem Cell Res.* 25, 50-60. doi:10.1016/j.scr.2017.10.012

Raciti, M., Granzotto, M., Duc, M. D., Fimiani, C., Cellot, G., Cherubini, E. and Mallamaci, A. (2013). Reprogramming fibroblasts to neural-precursor-like cells by structured overexpression of pallial patterning genes. *Mol. Cell. Neurosci.* 57, 42-53. doi:10.1016/j.mcn.2013.10.004

Spigoni, G., Gedressi, C. and Mallamaci, A. (2010). Regulation of Emx2 expression by antisense transcripts in murine cortico-cerebral precursors. *PLoS ONE* 5, e8658. doi:10.1371/journal.pone.0008658
