# Supplementary material for: Foxg1 bimodally tunes L1-mRNA and -DNA dynamics in the developing murine neocortex
Source: Development. 2024 May 23;151(10):dev202292. doi: 10.1242/dev.202292 (PMC11190451; doi:10.1242/dev.202292)
Supplement: Supplementary file 1 [file develop-151-202292_review_history.pdf]

Foxg1 bimodally tunes L1-mRNA and -DNA dynamics in the developing murine neocortex

Gabriele Liuzzi, Osvaldo Artimagnella, Simone Frisari and Antonello Mallamaci
DOI: 10.1242/dev.202292

Editor: Francois Guillemot

Review timeline

Original submission:	23 August 2023
Editorial decision:	29 September 2023
First revision received:	22 March 2024
Editorial decision:	8 April 2024
Second revision received:	13 April 2024
Accepted:	16 April 2024

Original submission

First decision letter

MS ID#: DEVELOP/2023/202292

MS TITLE: Foxg1 bimodally tunes L1-mRNA and -DNA dynamics in the developing murine neocortex

AUTHORS: Gabriele Liuzzi and Antonello Mallamaci

I have now received the reports of three referees on your manuscript and I have reached a decision. The referees' comments are appended below, or you can access them online: please go to BenchPress and click on the 'Manuscripts with Decisions' queue in the Author Area.

As you will see, all the referees express great interest in your work, but they also have significant criticisms and recommend a substantial revision of your manuscript before we can consider publication. In particular, referees 1 and 2 comment on the difficulty to read the manuscript and request that the manuscript is rewritten in a clearer and simpler manner. If you are able to revise the manuscript along the lines suggested, which may involve further experiments, I will be happy to receive a revised version of the manuscript. Your revised paper will be re-reviewed by one or more of the original referees, and acceptance of your manuscript will depend on your addressing satisfactorily the reviewers' major concerns. Please also note that Development will normally permit only one round of major revision. If it would be helpful, you are welcome to contact us to discuss your revision in greater detail. Please send us a point-by-point response indicating your plans for addressing the referees' comments, and we will look over this and provide further guidance.

Please attend to all of the reviewers' comments and ensure that you clearly highlight all changes made in the revised manuscript. Please avoid using 'Tracked changes' in Word files as these are lost in PDF conversion. I should be grateful if you would also provide a point-by-point response detailing how you have dealt with the points raised by the reviewers in the 'Response to Reviewers' box. If you do not agree with any of their criticisms or suggestions please explain clearly why this is so.

Reviewer 1

© 2023. Published by The Company of Biologists under the terms of the Creative Commons Attribution License (https://creativecommons.org/licenses/by/4.0/).

Advance summary and potential significance to field

In their manuscript, Liuzzi and Mallamaci investigate the interactions between Foxg1 and transposable elements of the L1 family. The authors show that Foxg1 protein can bind to L1 elements and repress their transcription by controlling epigenetic modifications specifically in neurogenic progenitors and postmitotic neurons. Accordingly, Foxg1 loss of function and overexpression result in increased and reduced L1 mRNA expression, respectively. Interestingly, Foxg1 also affects L1 DNA copy numbers, though in the opposite direction. It does so by interfering with the interaction between L1 mRNA and the helicases Mov10 and Ddx39a that are required for L1 retrotranscription. Based on these findings, the authors conclude that Foxg1 acts in a bimodal way to fine-tune L1 retrotransposition.
Foxg1 is a master regulator of telencephalic development and its mutations result in severe neurodevelopmental disorders. Hence, it is crucial to obtain a fuller understanding of its function. This manuscript sheds light on a Foxg1 role beyond the transcriptional regulation of coding genes and also suggests a novel role in non-transcriptional control of RNA biology.

Comments for the author

There are a number of points I would like the authors to clarify:

Major points:
1. The authors use various manipulations including Crispr/Cas9 gene editing, shRNA mediated knock-down and overexpression to alter Foxg1 expression levels. While they indicate Foxg1 mRNA levels in Supplementary Table 1, they do not provide information on protein levels. Furthermore, the overexpression of Foxg1W308X (14fold!) appears non-physiological and is likely to lead to artefacts.

2. The authors use a sophisticated three tier culture system to evaluate Foxg1 function in specific cell populations. These cultures contain various mixtures of neural stem cells, neural progenitors and neurons whose composition, moreover, changes with Foxg1 manipulation. Hence, the interpretation of the author's findings becomes very complex and therefore requires further experimental validation. For example, specific cell populations could be FACS sorted before subsequent analysis.

3. The physiological significance of Foxg1 mediated control of L1 transcription and DNA copy number remains unclear. Although this is certainly difficult to test experimentally, the authors could extend their discussion which appears too general at this stage.

4. Figure 6A: In protocol I cultures mainly containing neural stem cells, Foxg1 protein does not bind to L1 DNA under physiological conditions, all p values are larger than 0.05; significant binding only occurs after Foxg1 overexpression. Although eventually stated, it needs to be made clearer in the text that Foxg1 probably does not act in this cell type to control L1 transcription. In general, the manuscript lacks clarity throughout and is often very difficult to read. Proof-reading by a native English speaker might be beneficial to better convey the important message of this manuscript.

5. Figure 9: In experiments to address the basis of Foxg1 induced epigenetic changes at L1 elements, the authors use the truncated Foxg1W308X protein lacking binding domains for Groucho and KDM5B. This is an excellent control but it needs to be supported by including a Foxg1 wild-type control in this specific set of experiments. It is not sufficient to refer to earlier, independent experiments.

6. On a number of occasions, p values larger than 0.05 were interpreted to indicate significant changes, which they don't. The authors need to take a more balanced view throughout the manuscript.

Minor points:
1. Some of the p values do not correspond between the main text and the figures; for example at the end of the first paragraph in the section entitled "Modeling Foxg1 regulation of L1-mRNA" or page 6 for Figure 3G.

© 2023. Published by The Company of Biologists under the terms of the Creative Commons Attribution License (https://creativecommons.org/licenses/by/4.0/).

2. The reason why different methods of normalisation were used is not explained sufficiently. Normalisation should be handled more consistently throughout the text.

Reviewer 2

Advance summary and potential significance to field

The transcription factor Foxg1 plays multiple roles during embryonic forebrain development and its functions are likely to be of broad interest to readers of Development. This manuscript comes from a lab with a long track record of interesting and important discoveries concerning Foxg1's normal functions.

The manuscript convincingly demonstrates that Foxg1 regulates expression and transposition activity of L1 elements in embryonic mouse brain cells, a novel and potentially interesting finding, significantly adding to our understanding of the mechanisms by which Foxg1 controls forebrain development.

Comments for the author

Unfortunately, I found the manuscript incredibly difficult to follow. At heart, the experimental approach taken is straightforward – Foxg1 was overexpressed in a variety of forebrain cells in a variety of contexts and the effects on L1 were determined. However, the findings are presented in an unnecessarily complicated manner that makes them very difficult to understand. I would suggest that the manuscript be rewritten in a simpler way, to convey the novel findings clearly, such that they can be readily understood by readers. As a simple example, including a diagram to illustrate the relative positions of the various regions of L1 that were used for qPCR and adding a sentence of biological explanation as to why these specific regions were chosen would have been very helpful. As a further example, at the bottom of page 6, the authors say that "Foxg1 recruitment at L1 loci can be negligible in NSCs and significant in NPs" but this is very difficult to work out from Fig 6, because the extensive labelling within the figure doesn't directly indicate NSCs or NPs and the reader has to work this out from other information in the figure.

The experiments are thorough and include clear evidence that increasing or decreasing levels of Foxg1 have opposite effects on L1 expression. I think that it's important to sound a note of caution when interpreting the results of overexpression studies. In this case, wild type cells are used, which already contain normal levels of Foxg1. Therefore, overexpression leads to the production of non-physiological levels of the protein, which may therefore behave in ways that it would normally do when present within its normal physiological boundaries. This is particularly important for proteins such as Foxg1, whose activities have been shown to depend on expression levels (eg doi: 10.1016/j.neuron.2012.04.025.). In the present case, the reservation is tempered somewhat by the clear evidence that decreased levels of Foxg1 have the opposite effect of overexpression and the inclusion of a patient-specific mutation of Foxg1, but I think that the authors should at least mention this caveat in their discussion. Also in the discussion, the authors could include a bit more about the possible biological significance of Foxg1's effects on L1 expression and retrotransposition – how might this fit with the large body of knowledge we already have concerning Foxg1's roles in forebrain development?

Page 10: authors say that Foxg1 mRNA levels are reduced by ~33% in het mutants, but what about protein levels? (maybe unknown, but definitely important and don't necessarily mirror mRNA levels)
Finally, I think that the manuscript would benefit from careful and thorough copy-editing, as there are a number of problems with the English.

Minor points:
'articulation' is used in a number of places in the manuscript, but I do not understand what the authors mean by this term (eg line 1 of abstract)
Fig. 3 – the panels are labelled out of alphabetical order
Several figures consist of one or only a few panels and could readily be combined to make larger figures.
Page 12 'Propedeutically' – this word in not in the OED, I have no idea what it means

© 2023. Published by The Company of Biologists under the terms of the Creative Commons Attribution License (https://creativecommons.org/licenses/by/4.0/).

Reviewer 3

Advance summary and potential significance to field

Gabriele Liuzzi et al. have conducted an investigation into the role of Foxg1 in controlling the development of a specific region of the brain, the telencephalon.
Their research indicates that Foxg1 has the potential to regulate L1 at both DNA and RNA levels. L1 is a significant gene due to its expression in the central nervous system (CNS), and it can influence the brain's capacity to modify its genetic information. Furthermore, Foxg1 can either inhibit or promote L1 activity in various brain cells. This finding is noteworthy as it represents the first instance of a gene having a dual role in shaping the brain.

Comments for the author

However, there are several concerns that need to be addressed in this paper. At present, the manuscript is not suitable for publication due to the following major comments:

Identification of Neuron Progenitor Cells: In Figure 2, the authors use Sox2- to identify neuron progenitor cells. However, it is important to clarify why Sox2- cells can be identified as neural progenitor cells or provide references to support this assertion.

Analysis of ChIP-Seq Data: The authors performed ChIP-Seq experiments but did not explain their data analysis methods in detail. The manuscript mentions only that results were evaluated using Student's t-test via Excel software. A comprehensive description of the data analysis pipeline, including the software and reference genome used, is necessary.

Figure 3A Significance: In Figure 3A, the text mentions significant changes in percentages, but these changes are not reflected in the figure. The significance values should be visible in the graph, and the discrepancies need clarification.

Figure 3B: The manuscript introduces Plap-OE without explanation, and it is unclear if the authors successfully restricted Foxg1 expression in different cell types as mentioned. Additionally, the impact of Foxg1 introduction on cell proportion changes should be explained.

Figure 5: The identification of cell types in Figure 5 is based solely on Tubb3 whereas Figure 2 combined Tubb3 with Sox2. The rationale for this difference in marker genes should be addressed.

Figure 6: When discussing the mechanism of how Foxg1 controls L1 expression in Figure 6, it is suggested that ChIP-Seq data were used. However, there is a lack of bioinformatic analysis or visualization of differently enriched peaks between Foxg1 overexpression and control. The use of CRISPR knockout on Foxg1 for further mechanistic insights is also suggested.

Figures 10, 11, and 12: The concept of "cumulative L1 copy number" should be clarified and supported by Sanger sequencing results. These results should also be explained in Figures 11 and 12.

L1 mRNA Dynamics: The increase in L1 mRNA in Foxg1+/- mutants in Figure 1 is not robust, and the reasons for this inconsistency should be addressed.

Microscopic Images in Fig 2: Magnified representative microscopic images in Figure 2 are necessary, as the brightfield images do not appear to align with the immunofluorescence images.

Opposite L1 mRNA Dynamics: Figure 2C shows opposing L1 mRNA dynamics from E14.5 to P0 in the mesencephalic tectum, and the reasons for this should be discussed.

Figure 3E-F: In the mid-neurogenic culture, the decrease in L1 mRNA when Foxg1 is overexpressed is minor. It is suggested that using RNAscope and co-staining with cell-type-specific markers could provide more promising results.

© 2023. Published by The Company of Biologists under the terms of the Creative Commons Attribution License (https://creativecommons.org/licenses/by/4.0/).

ChIP Assay: Performing ChIP assays with FOXG1 and FOXG1W308X mutant followed by qPCR evaluation of diagnostic amplicons (Orf2, 5'UTR.A, 5'UTR.Tf) would strengthen the claim that Foxg1 binds to the L1 mRNA promoter to regulate transcription.

Figure 11: In the mesencephalic tectum, L1 DNA copy number increases upon Foxg1 overexpression from E14.5 to P0, but the corresponding L1 mRNA transcript levels should also be examined.

Inconsistent Observations: The paper reports that in the absence of Foxg1, L1 DNA copy number is reduced, while L1 mRNA expression is increased. This inconsistency needs to be addressed and explained.

Additionally, there are some minor comments:
L1 Genomic Regions: The meaning of "L1.orf2" and "L1.5'UTR.A, .Gf, and .Tf" should be clarified and supported by references. Data presentation in Figure 1 could be improved.
DIV Abbreviation: The abbreviation "DIV" should be explained earlier in the manuscript. It should also be clarified how specific time points like DIV0.87 were determined.
Fig2B 'Mes' Box: The significance of the grey box labeled 'mes' in Figure 2B should be explained.
Addressing these major and minor comments will significantly enhance the clarity, reliability, and completeness of the manuscript, making it more suitable for publication.

First revision

Author response to reviewers' comments

Responses to Reviewer 1

Advance Summary and Potential Significance to Field:
In their manuscript, Liuzzi and Mallamaci investigate the interactions between Foxg1 and transposable elements of the L1 family. The authors show that Foxg1 protein can bind to L1 elements and repress their transcription by controlling epigenetic modifications specifically in neurogenic progenitors and postmitotic neurons. Accordingly, Foxg1 loss of function and overexpression result in increased and reduced L1 mRNA expression, respectively. Interestingly, Foxg1 also affects L1 DNA copy numbers, though in the opposite direction. It does so by interfering with the interaction between L1 mRNA and the helicases Mov10 and Ddx39a that are required for L1 retrotranscription. Based on these findings, the authors conclude that Foxg1 acts in a bimodal way to fine-tune L1 retrotransposition.
Foxg1 is a master regulator of telencephalic development and its mutations result in severe neurodevelopmental disorders. Hence, it is crucial to obtain a fuller understanding of its function. This manuscript sheds light on a Foxg1 role beyond the transcriptional regulation of coding genes and also suggests a novel role in non-transcriptional control of RNA biology.

Reviewer 1 Comments for the Author:
There are a number of points I would like the authors to clarify:

Major points:

1. The authors use various manipulations including Crispr/Cas9 gene editing, shRNA mediated knock- down and overexpression to alter Foxg1 expression levels. While they indicate Foxg1 mRNA levels in Supplementary Table 1, they do not provide information on protein levels. Furthermore, the overexpression of Foxg1W308X (14fold!) appears non-physiological and is likely to lead to artefacts.

To evaluate Foxg1 protein levels upon gene manipulation, we measured such levels in as many as 6

© 2023. Published by The Company of Biologists under the terms of the Creative Commons Attribution License (https://creativecommons.org/licenses/by/4.0/).

different, gain- and loss-of-function configurations, corresponding to experiments described in Fig. 1&13, 3(a), 4(3), 5A, 5B, and 5C, and reported the results in new Fig. S2

Concerning potential risks posed by 14x FOXG1(W308X) overexpression, Frisari et al (2022) showed that an even more pronounced overexpression of the same allele in similar, neuron-enriched primary cultures did elicit trans-activating or trans-repressing effects similar to those played by the wt allele (on (a) Arc, Hes1, Npas4, Grik3, Grik4, Cacna2d2, Scn11a and Grin2c, and (b) endo-Foxg1, Gad1, Gad2, Gabra1, respectively), albeir less pronounced. This rules out the occurrence of artifactual dominant-negative effects.

2. The authors use a sophisticated three tier culture system to evaluate Foxg1 function in specific cell populations. These cultures contain various mixtures of neural stem cells, neural progenitors and neurons whose composition, moreover, changes with Foxg1 manipulation. Hence, the interpretation of the author's findings becomes very complex and therefore requires further experimental validation. For example, specific cell populations could be FACS sorted before subsequent analysis.

As suggested, we FACsorted (a) NSCs and (b) NPs+Ns from neocortical neural preparations made loss-of-function for Foxg1 via Cas9-KD, and we demonstrated that - consistently with previously argued - natural Foxg1-driven downregulation of mL1-mRNA is restricted to NPs+Ns only. Results of this novel assay are illustrated in new Fig. 8.

3. The physiological significance of Foxg1 mediated control of L1 transcription and DNA copy number remains unclear. Although this is certainly difficult to test experimentally, the authors could extend their discussion which appears too general at this stage.

After the initial submission of our manuscript to Development, a major paper, by Mangoni et al (2023), was published, reporting an investigation of the impact of L1 knock-down on neocortical development.

The intersection among the phenotype described by Mangoni et al and previous literature on Foxg1 control of neocorticogenesis suggested us that repression of L1-mRNA might contribute to mediate Foxg1 control over specific aspects of neocortical histogenesis (neuronogenic NSC commitment, radial neuronal migration and astrogenesis progression). These considerations were included into the revised Discussion of the manuscript, at page 20, row 17.

4. Figure 6A: In protocol I cultures mainly containing neural stem cells, Foxg1 protein does not bind to L1 DNA under physiological conditions, all p values are larger than 0.05; significant binding only occurs after Foxg1 overexpression. Although eventually stated, it needs to be made clearer in the text that Foxg1 probably does not act in this cell type to control L1 transcription. In general, the manuscript lacks clarity throughout and is often very difficult to read. Proof-reading by a native English speaker might be beneficial to better convey the important message of this manuscript.

For the sake of clarity, the conclusions of the two first periods of page 11, illustrating these concepts, were reprhased, as follows:
at row 5, "Taking into account the high prevalence of NSCs in early ("*prot I*"-type) Plap-OE cultures and their massive conversion into NPs elicited by Foxg1-OE (as shown in Fig. 5A (1)), ChIP results shown in Fig. 6A *might* reflect selective Foxg1 recruitment at mL1 loci in NPs, but not in NSCs (Fig. 7 (1)).

[......]

at row 12, "Together with the high prevalence of NPs and Ns in all "*prot II*"-type cultures (as shown Fig. 5C (1)), this scenario points to Foxg1 binding to L1 loci in NPs and/or Ns (Fig. 7)."

As for general clarity issues raised by Reviewer 1, we think they may have largely stemmed from complexity of results and inference chains needed for their interpretation. We did our best to improve clarity, while preserving rigor of logical reasoning. Moreover, we carefully revised the

© 2023. Published by The Company of Biologists under the terms of the Creative Commons Attribution License (https://creativecommons.org/licenses/by/4.0/).

language.

5. Figure 9: In experiments to address the basis of Foxg1 induced epigenetic changes at L1 elements, the authors use the truncated Foxg1W308X protein lacking binding domains for Groucho and KDM5B. This is an excellent control but it needs to be supported by including a Foxg1 wild-type control in this specific set of experiments. It is not sufficient to refer to earlier, independent experiments.

We replaced the old Fig. 9 with the new Fig. 10, where the impact of Plap, FOXG1(WT) and FOXG1(W308X) on mL1-mRNA levels was comparatively co-evaluated over the E16.5 + DIV8 window. Old Fig. 9 results were moved to Fig. S3.

6. On a number of occasions, p values larger than 0.05 were interpreted to indicate significant changes, which they don't. The authors need to take a more balanced view throughout the manuscript.

As suggested, we carefully revised the text, avoiding to interpret p values exceeding 0.05 as an index of formal significance. In these cases, only when p got close to such threshold, we referred to an *increasing or decreasing trend* of the parameter investigated.

Minor points:

1. Some of the p values do not correspond between the main text and the figures; for example at the end of the first paragraph in the section entitled "Modeling Foxg1 regulation of L1-mRNA" or page 6 for Figure 3G.

Text and figures were co-scanned for p congruence and inconsistencies were fixed.

2. The reason why different methods of normalisation were used is not explained sufficiently. Normalisation should be handled more consistently throughout the text.

qRTPCR evaluation of gene expression levels usually relies on the employment of a defined normalizer supposed to exhibit stable absolute expression across the experimental conditions considered (Gapdh, Rlp10a among those). The systematic use of multiple normalizers would be obviously auspicable, however it would pose serious issues as for its practical affordability.To deal with these two opposite constraints, we employed normalization against multiple genes, however restricting it to only a subset of key assays.

Responses to Reviewer 2

Advance Summary and Potential Significance to Field:
The transcription factor Foxg1 plays multiple roles during embryonic forebrain development and its functions are likely to be of broad interest to readers of Development. This manuscript comes from a lab with a long track record of interesting and important discoveries concerning Foxg1's normal functions.

The manuscript convincingly demonstrates that Foxg1 regulates expression and transposition activity of L1 elements in embryonic mouse brain cells, a novel and potentially interesting finding, significantly adding to our understanding of the mechanisms by which Foxg1 controls forebrain development.

Reviewer 2 Comments for the Author:

© 2023. Published by The Company of Biologists under the terms of the Creative Commons Attribution License (https://creativecommons.org/licenses/by/4.0/).

Unfortunately, I found the manuscript incredibly difficult to follow. At heart, the experimental approach taken is straightforward – Foxg1 was overexpressed in a variety of forebrain cells in a variety of contexts and the effects on L1 were determined. However, the findings are presented in an unnecessarily complicated manner that makes them very difficult to understand. I would suggest that the manuscript be rewritten in a simpler way, to convey the novel findings clearly, such that they can be readily understood by readers. As a simple example, including a diagram to illustrate the relative positions of the various regions of L1 that were used for qPCR and adding a sentence of biological explanation as to why these specific regions were chosen would have been very helpful.

As for clarity issues, we think they may have largely stem from complexity of results and inference chains needed for their interpretation. We did our best to improve clarity, while preserving rigor of logical reasoning. Moreover, we carefully revised the language.

In particular, as for the example mentioned above, we prepared a dedicated graph with the location of the diagnostic amplicons employed (reported in new Fig. S1), as well as a synopsis of their Sanger- sequencing validation (reported in the new Table S1).
Fig. S1 and Table S1, as well as two key references providing fundamental information about taxonomy and anatomy of murine LINE1 elements, were cited upon first mention of such amplicons, at page 6, row 9

As a further example, at the bottom of page 6, the authors say that "Foxg1 recruitment at L1 loci can be negligible in NSCs and significant in NPs" but this is very difficult to work out from Fig 6, because the extensive labelling within the figure doesn't directly indicate NSCs or NPs and the reader has to work this out from other information in the figure.

To improve the clarity of the message, the text referring to this issue (at page 11, row 5) was revised ed edited as follows:

"Taking into account the high prevalence of NSCs in early ("prot I"-type) Plap-OE cultures and their massive conversion into NPs elicited by Foxg1-OE (as shown in Fig. 5A (1)), ChIP results shown in Fig. 6A might reflect selective Foxg1 recruitment at mL1 loci in NPs, but not in NSCs (Fig. 7 (1)).
Next, we run similar assays on chromatin of mid-neuronogenic ("prot II"-type) cultures. In this case, a net Foxg1-enrichment was detectable at almost all diagnostic amplicons in Plap-OE controls ($p_{5'UTR.A}<0.02$, $p_{5'UTR.Gf}<0.09$, $p_{5'UTR.Tf}<0.04$, $p_{orf2}<0.02$, $p_{3'UTR}<0.02$, with n=5,5 or - case Gf - n=5,4) (Fig. 6B). Together with the high prevalence of NPs and Ns in all "prot II"-type cultures (as shown Fig. 5C (1)), this scenario points to Foxg1 binding to L1 loci in NPs and/or Ns (Fig. 7).
Moreover, extensive statistical annotations on Fig. 6 were simplified".

Finally, to secure our interpretation of these results, we run an additional assay, by which we proved that Foxg1-driven repression of mL1 takes physiologically place in NPs and Ns, but not in NSCs.
Results of this assays were illustrated in new Figure 8 and described at page 11, row 15.

The experiments are thorough and include clear evidence that increasing or decreasing levels of Foxg1 have opposite effects on L1 expression. I think that it's important to sound a note of caution when interpreting the results of overexpression studies. In this case, wild type cells are used, which already contain normal levels of Foxg1. Therefore, overexpression leads to the production of non- physiological levels of the protein, which may therefore behave in ways that it would normally do when present within its normal physiological boundaries. This is particularly important for proteins such as Foxg1, whose activities have been shown to depend on expression levels (eg doi: 10.1016/j.neuron.2012.04.025.). In the present case, the reservation is tempered somewhat by the clear evidence that decreased levels of Foxg1 have the opposite effect of overexpression and the inclusion of a patient-specific mutation of Foxg1, but I think that the authors should at least mention this caveat in their discussion.

As suggested, we added a brief paragraph to the Discussion (at page 18, row 9), stating:

© 2023. Published by The Company of Biologists under the terms of the Creative Commons Attribution License (https://creativecommons.org/licenses/by/4.0/).

"As a general note of methodological caution, the interpretation of results originating from gene overexpression-assays requires a special attention, because of possible occurrence of paradoxical dominant-negative effects, evoked by over-abundance of gene products. In this respect, it is worthy mentioning that Foxg1 expression gains peculiar to our assays usually fell below 5x, at both mRNA and protein levels (Table S2, and Fig. S2). More importantly, phenotypes evoked by Foxg1 overexpression generally mirrored those elicited by gene knock-down (see, for example, Fig. 3A vs Fig. 3B-G, or Fig. 4(2) vs Fig. 4(3)), suggesting that they provide a qualitatively genuine representation of the physiological functions exerted by Foxg1 in natural contexts."

Also in the discussion, the authors could include a bit more about the possible biological significance of Foxg1's effects on L1 expression and retrotransposition – how might this fit with the large body of knowledge we already have concerning Foxg1's roles in forebrain development?

After the initial submission of our manuscript to Development, a major paper, by Mangoni et al (2023), was published, reporting an investigation of the impact of L1 knock-down on neocortical development. The intersection among the phenotype described by Mangoni et al and previous literature on Foxg1 control of neocorticogenesis suggested us that repression of L1-mRNA might contribute to mediate Foxg1 control over specific aspects of neocortical histogenesis (neuronogenic NSC commitment, radial neuronal migration and astrogenesis progression). These considerations were included into the revised Discussion of the manuscript, at page 20, row 17.

Page 10: authors say that Foxg1 mRNA levels are reduced by ~33% in het mutants, but what about protein levels? (maybe unknown, but definitely important and don't necessarily mirror mRNA levels)

We evaluated Foxg1 protein levels by quantitative immunofluorescence, upon six different, loss- and gain-of-function, manipulations proposed for this study and we reported the results in the new Figure S2.

Finally, I think that the manuscript would benefit from careful and thorough copy-editing, as there are a number of problems with the English.

The text was carefully revised.

Minor points:
'articulation' is used in a number of places in the manuscript, but I do not understand what the authors mean by this term (eg line 1 of abstract)

Depending on contexts, improperly used "articulation" was replaced by more appropriate words (progression, implementation, occurrence.)

Fig. 3 – the panels are labelled out of alphabetical order

We ask Reviewer 2 to accept the unusual order along which panels are aligned in Figure 3, while complying with the rule to label panels according to their citation order within the main text, it eases comparison of D,E,F panels histograms, thanks to their shared vertical alignment.

Several figures consist of one or only a few panels and could readily be combined to make larger figures.

We ask the Reviewer to allow us not to merge small (mono-panel) Figures with others, as referring to distinctive conceptual blocks

© 2023. Published by The Company of Biologists under the terms of the Creative Commons Attribution License (https://creativecommons.org/licenses/by/4.0/).

Page 12 'Propedeutically' - this word in not in the OED, I have no idea what it means

We replaced 'Propedeutically' by 'Propaedeutically' ('Propaedeutical' is reported by both Collins and Oxford English Dictionary)

Responses to Reviewer 3

Advance Summary and Potential Significance to Field:
Gabriele Liuzzi et al. have conducted an investigation into the role of Foxg1 in controlling the development of a specific region of the brain, the telencephalon. Their research indicates that Foxg1 has the potential to regulate L1 at both DNA and RNA levels. L1 is a significant gene due to its expression in the central nervous system (CNS), and it can influence the brain's capacity to modify its genetic information. Furthermore, Foxg1 can either inhibit or promote L1 activity in various brain cells. This finding is noteworthy as it represents the first instance of a gene having a dual role in shaping the brain.

Reviewer 3 Comments for the Author:
However, there are several concerns that need to be addressed in this paper. At present, the manuscript is not suitable for publication due to the following major comments:

Identification of Neuron Progenitor Cells: In Figure 2, the authors use Sox2- to identify neuron progenitor cells. However, it is important to clarify why Sox2- cells can be identified as neural progenitor cells or provide references to support this assertion.

It has been shown that, within the neuronogenic pallium, high Sox2 expression spefically characterizes neural stem cells located in VZ, whereas null or very low Sox2 expression may be found in a subset of committed neuronogenic progenitors within the SVZ) (Hutton and Pveny, 2011). Actually, cells classified as Sox2(+) in our old Fig. 2A expressed Sox2 at high level, while those labeled as Sox2(-) expressed this protein at low or null level.

To make this point clear:

- at page 48, row 16, we added the sentence:
"to note, here, low-Sox2-expressing cells (<2*background) were classified as Sox2-, whereas all cells classified as Sox2+ expressed such protein at much higher levels (>5*background);

- at page 6, row 19, we added the sentence:
"Neural cells generated by these protocols were categorized based on their Sox2/Tubb3 expression profiles (Hutton and Pevny, 2011; Menezes and Luskin, 1994)".

Analysis of ChIP-Seq Data: The authors performed ChIP-Seq experiments but did not explain their data analysis methods in detail. The manuscript mentions only that results were evaluated using Student's t-test via Excel software. A comprehensive description of the data analysis pipeline, including the software and reference genome used, is necessary.

Data referred to in Fig. 6 and new Fig. 9 originated from simple ChIP-qPCR assays tailored against L1 amplicons (detailed in novel Figure S1 and Table S1). No systematic ChIP-Seq analysis was run.

Figure 3A Significance: In Figure 3A, the text mentions significant changes in percentages, but these changes are not reflected in the figure. The significance values should be visible in the graph, and the discrepancies need clarification.

At page 7, row 27, we replaced the sentence:
" Consistently with P0 Foxg1-/+ pups (Fig. 1), neocortical Foxg1-LOF cultures displayed a systematic

© 2023. Published by The Company of Biologists under the terms of the Creative Commons Attribution License (https://creativecommons.org/licenses/by/4.0/).

upregulation of L1-mRNAs, +25.9±7.4% ($p<0.004$), +21.6±10.9% ($p<0.054$), and +18.7±6.7% ($p<0.017$), as for sub-families A, Gf and Tf, respectively, with n=10,11 (Fig. 3A, S1A)." by the new text:
"Consistently with P0 Foxg1-/+ pups (Fig. 1) and upon normalization against the "Gapdh, Rpl10a, Cltc and rDNA18S quadruplet", neocortical Foxg1-LOF cultures displayed an increasing trend in L1-mRNAs originating from A, Gf and Tf families, which - however - did not reach statistical significance (Fig. 3A). [Significance was achieved upon normalization against Gapdh: +25.9±7.4% ($p<0.004$), +21.6±10.9% ($p<0.054$), and +18.7±6.7% ($p< 0.017$), respectively, with n=10,11 (Fig. S4A)]"

Figure 3B: The manuscript introduces Plap-OE without explanation, and it is unclear if the authors successfully restricted Foxg1 expression in different cell types as mentioned. Additionally, the impact of Foxg1 introduction on cell proportion changes should be explained.

At page 8, row 6, upon our first mention of overexpression assays, we added the sentence: "In these assays we employed the ALPP gene, encoding for human Placental alkaline phosphatase (hereafter referred to as Plap) as a control (Falcone et al., 2016)".

The pNes promoter driving transgene expression referred to in Fig. 3B is specifically active in NSCs, however we suspected that the upregulation of the protein encoded by it could persist in more mature derivatives of these cells. This has to be taken into account for a proper interpretation of the phenotype evoked by such protein. To make this point clear, we mapped protein expression levels to distinct cell types of the engineered culture, as reported in the dedicated Fig. 5A. This exercise confirmed our suspicion, showing that pNes was able to elicit an upregulation of Foxg1 protein not restricted to NSCs, but also detectable in neuronal progenitors and postmitotic neurons.
Concerning the impact of Foxg1 introduction on cell proportions, it was described at page 9, row 25: "Within early-neuronogenic preparations, pNes-driven Foxg1 elicited a prominent increase of NPs (f_{NP}(Foxg1-GOF)=39.16±1.14% vs f_{NP}(ctrl)=11.93±2.58%, $p<10^{-5}$, n=6,5) at expenses of NSCs (f_{NSC}(Foxg1-GOF)=57.81±1.53% vs f_{NSC}(ctrl)=84.58±2.81%, $p<10^{-5}$, n=6,5)".

The presumptive biological meaning of this phenomenon was proposed at page 20, row 6: "On one side, Foxg1 exerts a complex impact on neuronogenesis progression, (1) stimulating the NSC-to-NP transition, ".

To note, this interpretation is fully consistent with what previously reported by ref 5.

Figure 5: The identification of cell types in Figure 5 is based solely on Tubb3, whereas Figure 2 combined Tubb3 with Sox2. The rationale for this difference in marker genes should be addressed.

Actually, to discriminate among different cell types in Figure 5, we relied on Tubb3 (selectively expressed in neurons), as well as on mCherry driven by the NP/N-specific pTa1 promoter. Given the absence of glial cell types in these preparations, "black" (i.e. mCherry- Tubb3-) cells of Figure 5 are equivalent to Sox2+ cells of Figure 2.

Figure 6: When discussing the mechanism of how Foxg1 controls L1 expression in Figure 6, it is suggested that ChIP-Seq data were used. However, there is a lack of bioinformatic analysis or visualization of differently enriched peaks between Foxg1 overexpression and control. The use of CRISPR knockout on Foxg1 for further mechanistic insights is also suggested.

As highlighted above, data referred to in Fig. 6 and new Fig. 9 originated from simple ChIP-qPCR assays tailored against L1 amplicons (detailed in novel Figure S1 and Table S1). No systematic ChIP-Seq analysis was run.

As for CRISPR gene inactivation, it was employed in four sets of loss-of-function assays, as illustrated in Fig. 3A, 8, 14, 15.

© 2023. Published by The Company of Biologists under the terms of the Creative Commons Attribution License (https://creativecommons.org/licenses/by/4.0/).

Figures 10, 11, and 12: The concept of "cumulative L1 copy number" should be clarified and supported by Sanger sequencing results. These results should also be explained in Figures 11 and 12.

Here "cumulative" refers to all L1 elements, including prevailing 5' truncated ones originating from somatic retrotransposition.
This was explained by a new sentence, at page 13, row 6:
"For this purpose, we relied on the diagnostic 3'UTR amplicon (Fig. S1, Table S1), present in all L1 repeats, including prevailing 5' truncated ones originating from somatic retrotransposition (Babushok et al., 2006)").

This explanation obviously applies also to subsequent text, referring to new Fig. 12 and 13 (corresponding to old Fig. 11 and 12, respectively)

Amplicons obtained by diagnostic primers employed in this study were cloned and Sanger-sequenced. Results of these controls were summarirized in novel Table S1

L1 mRNA Dynamics: The increase in L1 mRNA in Foxg1+/- mutants in Figure 1 is not robust, and the reasons for this inconsistency should be addressed.

Actually the increase in L1 mRNA in Foxg1+/- mutants reported in Figure 1 is statistically significant (with p spanning from <0.014 to 10^{-4}). Its moderate amplitude obviously suggests that even one functional Foxg1 copy is able to exert a robust inhibition of L1 mRNA expression, comparable with that peculiar to Foxg1-wt mice.

Microscopic Images in Fig 2: Magnified representative microscopic images in Figure 2 are necessary, as the brightfield images do not appear to align with the immunofluorescence images.

As required, we added magnifications of selected fields of the three immunofluorescences in Fig. 2A. Moreover, for the sake of clarity, we edited the Legend to Fig. 2 (A) as follows:

"Figure 2. In vitro modeling of L1-mRNA progression in murine developing neocortex. In (A), from left to right, shown are: [a] the three protocols (type I, II and III) employed to generate primary cultures, which model early, mid and late phases of neuronogenesis, respectively, and include neural cells terminally exposed to GFs, GFs and serum, and serum, respectively; [b] prevalences of distinctive cell types forming these cultures (type I cultures enriched in neural stem cells (NSCs, Sox2+Tubb3-) and neuronogenic progenitors (NPs, Sox2-Tubb3-), type II ones including comparable fractions of NSCs, NPs and neurons (Ns, Tubb3+), and type III ones highly enriched in Ns); to note, here, low-Sox2- expressing cells (<2*background) were classified as Sox2-, whereas all cells classified as Sox2+ expressed such protein at much higher levels (>5*background); [c] examples of primary data; these include: bright field pictures of living cultures, taken just before their terminal analysis, and dark field images of aSox2/aTubb3 immunofluorescences, performed upon culture dissociation and fixation.

Opposite L1 mRNA Dynamics: Figure 2C shows opposing L1 mRNA dynamics from E14.5 to P0 in the mesencephalic tectum, and the reasons for this should be discussed.

A dedicated sentence was added, at page 19, row 20:
"This might reflect an intrinsically different regulation of *L1* biology in telencephalon vs mesencephalon and/or a developmental heterochrony between these two structures."

Figure 3E-F: In the mid-neurogenic culture, the decrease in L1 mRNA when Foxg1 is overexpressed is minor. It is suggested that using RNAscope and co-staining with cell-type-specific markers could provide more promising results.

© 2023. Published by The Company of Biologists under the terms of the Creative Commons Attribution License (https://creativecommons.org/licenses/by/4.0/).

Actually, in one of the two cases mentioned (Fig. 3E; driving promoter: pTa1), albeit small, the decrease in L1-mRNA displayed by Foxg1-OE, mid-neurogenic cultures was statistically significant for all three family-specific amplicons.
In the other case, i.e. when Foxg1 was driven by the pSyn promoter (Fig. 3F), it is true that only a decreasing trend was detectable. However, when pSyn-driven Foxg1 overexpression was allowed to take place for a longer time (Fig. 3G), then L1-mRNA decrease became fully statistically significant. We do think that such positive correlation between the robusteness of the L1-mRNA decrease detected and the duration of the treatment triggering it is a good specificity index, nicely corroborating the biological plausibility of what observed.

ChIP Assay: Performing ChIP assays with FOXG1 and FOXG1W308X mutant followed by qPCR evaluation of diagnostic amplicons (Orf2, 5'UTR.A, 5'UTR.Tf) would strengthen the claim that Foxg1 binds to the L1 mRNA promoter to regulate transcription.

The study of FOXG1(W308X) impact on *L1*-mRNA levels was intended here as a simple specificity control. The systematic analysis of the epigenetic outcomes originating from the overexpression of mutant Foxg1 alleles is out of the scopes of this manuscript. It will be subject of a dedicated, future study.

Figure 11: In the mesencephalic tectum, L1 DNA copy number increases upon Foxg1 overexpression from E14.5 to P0, but the corresponding L1 mRNA transcript levels should also be examined.

We are perplexed. actually *L1*-mRNA transcript levels were also examined in mesencephalon, as reported in old Fig. 2C.

Inconsistent Observations: The paper reports that in the absence of Foxg1, L1 DNA copy number is reduced, while L1 mRNA expression is increased. This inconsistency needs to be addressed and explained.

Not consistent regulation of L1 mRNA and DNA copy number was commented at page 19, row 11, as follows:
" More in general, it has been shown/suggested that different products of L1 activity (RNA, DNA, orf1/2 proteins) have been highjacked to distinctive aspects of cell physiology (Blaudin De Thé et al., 2018; Chow et al., 2010; Madabhushi et al., 2015; Mangoni et al., 2023; Muotri and Gage, 2006), so obviously requiring differential tuning of their dosages. Thanks to its bimodal impact on L1 transcription and retrotranscription, Foxg1 might just contribute to such complex regulation."

Additionally, there are some minor comments:
L1 Genomic Regions: The meaning of "L1.orf2" and "L1.5'UTR.A, .Gf, and .Tf" should be clarified and supported by references. Data presentation in Figure 1 could be improved.

A map with the localization of each diagnostic amplicon was included in panel A of novel Fig. S1, and a validation of the corresponding primers was included in new Table S1.
Moreover two relevant key references (Sookdeo et al., 2013; Storer et al., 2021) were added upon first mention of these reagents, at page 6, row 11.

DIV Abbreviation: The abbreviation "DIV" should be explained earlier in the manuscript. It should also be clarified how specific time points like DIV0.87 were determined.

We revised the Materials and Methods section: we explained the DIV acronym upon its first usage (page 23, row 17) and replaced the old "DIV0.87" by the more usual "20 hours" (page 23, row 23).

© 2023. Published by The Company of Biologists under the terms of the Creative Commons Attribution License (https://creativecommons.org/licenses/by/4.0/).

Fig2B 'Mes' Box: The significance of the grey box labeled 'mes' in Figure 2B should be explained.

We removed this typo.

Addressing these major and minor comments will significantly enhance the clarity, reliability, and completeness of the manuscript, making it more suitable for publication.

We thank Reviewer 3 for his/her comments and suggestions.

Second decision letter

MS ID#: DEVELOP/2023/202292

MS TITLE: Foxg1 bimodally tunes L1-mRNA and -DNA dynamics in the developing murine neocortex

AUTHORS: Gabriele Liuzzi, Osvaldo Artimagnella, Simone Frisari, and Antonello Mallamaci

I have now received the reports of the three referees who reviewed the earlier version of your manuscript and I have reached a decision. The referees' comments are appended below, or you can access them online: please go to BenchPress and click on the 'Manuscripts with Decisions' queue in the Author Area.

The reviewers' overall evaluation is very positive and we would like to publish a revised manuscript in Development, provided that you satisfactorily address the remaining suggestions and comments of the referees. Please attend to all of the reviewers' comments in your revised manuscript and detail them in your point-by-point response. If you do not agree with any of their criticisms or suggestions explain clearly why this is so.

Reviewer 1

Advance summary and potential significance to field

In their manuscript, Liuzzi and Mallamaci investigate the interactions between Foxg1 and transposable elements of the L1 family. The authors show that Foxg1 protein can bind to L1 elements and repress their transcription by controlling epigenetic modifications specifically in neurogenic progenitors and postmitotic neurons. Accordingly, Foxg1 loss of function and overexpression result in increased and reduced L1 mRNA expression, respectively. Interestingly, Foxg1 also affects L1 DNA copy numbers, though in the opposite direction. It does so by interfering with the interaction between L1 mRNA and the helicases Mov10 and Ddx39a that are required for L1 retrotranscription. Based on these findings, the authors conclude that Foxg1 acts in a bimodal way to fine-tune L1 retrotransposition.
Foxg1 is a master regulator of telencephalic development and its mutations result in severe neurodevelopmental disorders. Hence, it is crucial to obtain a fuller understanding of its function. This manuscript sheds light on a Foxg1 role beyond the transcriptional regulation of coding genes and also suggests a novel role in non-transcriptional control of RNA biology.

Comments for the author

The authors addressed all my points but I have to admit the manuscript is still difficult to read. Scientific rigor is certainly important, but findings need to be communicated in an easily readable way for the broad audience of Development. There are also a few minor points which require amendments. Otherwise, I support publication of the manuscript in Development.

Minor points:

© 2023. Published by The Company of Biologists under the terms of the Creative Commons Attribution License (https://creativecommons.org/licenses/by/4.0/).

1) Statistical tests:
In Fig. 2B and Fig. 10 non-appropriate t-tests were used. As three experimental groups were compared, ANOVA tests should be performed.

2) There are still a couple of statistically non-significant results which are presented as significant in the main text
Fig 8: Npos + Ns for mLINE1.A; text on page 11 lines 22 and 23 needs to be adjusted.
Fig. 8: page 12 line 17: there is only a trend for a decrease in H3K4me3 (by the way, it should probably read Figure 9)

3) Some p-values in Figures 2C; 3C; 3E; 5A; 12 do not match between figure and main text.

4) Typos
Page 8; line 9: overexpression

Reviewer 2

Advance summary and potential significance to field

The authors have convincingly shown evidence that the transcription factor Foxg1 regulates the retrotransposon L1, with potentially important biological significance for brain development.

Comments for the author

The authors have addressed all of the scientific concerns I raised about the first version of this manuscript.
the experiments presented are thorough and I found the results convincing.
Although the authors have made some small changes to the manuscript, marginally improving the comprehensibility of some sections, the manuscript overall remains very difficult to read and understand, not because of the complexity of the experiments or their outocmes, but because of the way it is written. I would respectfully suggest that the authors seek to improve the clarity of their manuscript, to help their interesting work reach a wide audience.

Reviewer 3

Advance summary and potential significance to field

This study demonstrates the novel role of Foxg1 in regulating telencephalic development by exerting pleiotropic control over its progression. It explores the expression of L1 retrotransposons within the central nervous system (CNS) and suggests their potential contribution to genomic plasticity. By identifying putative Foxg1 binding motifs within L1 elements, the study proposes that Foxg1 may restrict the expression and activity of L1 retrotransposons in the telencephalon. Through in vivo and primary neural culture experiments using loss- and gain-of-function approaches, the study confirms this prediction showing that Foxg1-dependent transcriptional repression of L1 occurs specifically in neopallial neuronogenic progenitors and post-mitotic neurons.
Additionally, the study reveals a physical interaction between Foxg1 and L1-mRNA, with Foxg1 positively impacting neonatal neopallium L1-DNA content.
Surprisingly, Foxg1 antagonizes the retrotranscription-suppressing activity of Mov10 and Ddx39a helicases on L1 elements. This study establishes Foxg1 as the first CNS patterning gene with bimodal regulation of retrotransposons, both limiting and promoting L1 transcription and amplification within a specific domain of the developing mouse brain.

Comments for the author

The authors have addressed most of my previous comments. However, they should revise the text to ensure their study is put in the context of the previously known literature of this context.
For example, this study significantly advances our understanding of neurodevelopmental processes by elucidating the intricate interplay between Foxg1 and L1 retrotransposons in the context of

© 2023. Published by The Company of Biologists under the terms of the Creative Commons Attribution License (https://creativecommons.org/licenses/by/4.0/).

telencephalic development. The findings shed light on the regulatory mechanisms governing genomic plasticity within the central nervous system (CNS), a crucial aspect of neurodevelopmental processes. By identifying Foxg1 as a key regulator of L1 retrotransposon activity through both transcriptional repression and promotion, the study highlights the multifaceted role of this transcription factor in shaping the molecular landscape of the developing brain.
Moreover, the study's emphasis on the specific localization of Foxg1-dependent L1 repression to neopallial neuronogenic progenitors and post-mitotic neurons underscores the importance of spatial and temporal regulation in neurodevelopment. This insight contributes to our broader understanding of how gene regulatory networks orchestrate regional patterning and cellular differentiation within the developing CNS.
Furthermore, the unexpected discovery of Foxg1's interaction with L1-mRNA and its role in modulating L1-DNA content adds a new dimension to our understanding of retrotransposon regulation in neurodevelopment. By revealing Foxg1's ability to counteract the suppressive activities of Mov10 and Ddx39a helicases on L1 elements, the study highlights the complexity of the regulatory networks involved in retrotransposon biology.
How these aspects are related to previous findings of transcription factors retrotransposons and cell-fate needs to be properly highlgihted.

Second revision

Author response to reviewers' comments

Responses to Reviewer 1

Reviewer 1 Advance Summary and Potential Significance to Field:
In their manuscript, Liuzzi and Mallamaci investigate the interactions between Foxg1 and transposable elements of the L1 family. The authors show that Foxg1 protein can bind to L1 elements and repress their transcription by controlling epigenetic modifications specifically in neurogenic progenitors and postmitotic neurons. Accordingly, Foxg1 loss of function and overexpression result in increased and reduced L1 mRNA expression, respectively. Interestingly, Foxg1 also affects L1 DNA copy numbers, though in the opposite direction. It does so by interfering with the interaction between L1 mRNA and the helicases Mov10 and Ddx39a that are required for L1 retrotranscription. Based on these findings, the authors conclude that Foxg1 acts in a bimodal way to fine-tune L1 retrotransposition.
Foxg1 is a master regulator of telencephalic development and its mutations result in severe neurodevelopmental disorders. Hence, it is crucial to obtain a fuller understanding of its function. This manuscript sheds light on a Foxg1 role beyond the transcriptional regulation of coding genes and also suggests a novel role in non-transcriptional control of RNA biology.

We thank Reviewer 1 for her/his appreciation of our work.

Reviewer 1 Comments for the Author:
The authors addressed all my points but I have to admit the manuscript is still difficult to read. Scientific rigor is certainly important, but findings need to be communicated in an easily readable way for the broad audience of Development. There are also a few minor points which require amendments. Otherwise, I support publication of the manuscript in Development.

We further edited the entire text, in both lexicon and syntax, aiming to make it more understandable. We also largely repharased the "RESULTS, Modeling Foxg1 regulation of L1-mRNA" section (from page 9, row 18, to page 10, row 28), trying to accompany the reader through complex correlative evidence which points to selective L1-mRNA downregulation in neuronogenic progenitors. Finally, we reorganized the last part of the DISCUSSION (from page 18, row 18, to end), trying to simplify its logical architecture and make it more readable.

© 2023. Published by The Company of Biologists under the terms of the Creative Commons Attribution License (https://creativecommons.org/licenses/by/4.0/).

Minor points:

1) Statistical tests:

In Fig. 2B and Fig. 10 non-appropriate t-tests were used. As three experimental groups were compared, ANOVA tests should be performed.

The ANOVA test would provide us with a general estimate of the probability that the independent variable in order controls the supposed "dependent" variable. Actually, in cases of both Fig. 2B and Fig.10, we were not interested in assessing such probability. Conversely, we wanted to simply ascertain if statistically significant differences occurred between the states of the "dependent variable" associated to two specific states of the independent variable. For this reason, we intentionally employed a simple t-test in place of one-way ANOVA.

2) There are still a couple of statistically non-significant results which are presented as significant in the main text

Fig 8: Npos + Ns for mLINE1.A; text on page 11 lines 22 and 23 needs to be adjusted.

Fig. 8: page 12 line 17: there is only a trend for a decrease in H3K4me3 (by the way, it should probably read Figure 9)

The text was fixed according to Reviewer 1 suggestion

3) Some p-values in Figures 2C; 3C; 3E; 5A; 12 do not match between figure and main text.

We systematically checked text and figures against primary data, we remade calculations and, where appropriate, we edited Figures as due.

4) Typos

Page 8; line 9: overexpression

Typo was fixed.

Responses to Reviewer 2

Reviewer 2 Advance Summary and Potential Significance to Field:

The authors have convincingly shown evidence that the transcription factor Foxg1 regulates the retrotransposon L1, with potentially important biological significance for brain development.

Reviewer 2 Comments for the Author:

The authors have addressed all of the scientific concerns I raised about the first version of this manuscript. the experiments presented are thorough and I found the results convincing.

We thank Reviewer 2 for her/his appreciation of our work.

Although the authors have made some small changes to the manuscript, marginally improving the comprehensibility of some sections, the manuscript overall remains very difficult to read and understand, not because of the complexity of the experiments or their outocmes, but because of the way it is written. I would respectfully suggest that the authors seek to improve the clarity of their manuscript, to help their interesting work reach a wide audience.

We further edited the entire text, in both lexicon and syntax, aiming to make it more understandable. We also largely repharased the "RESULTS, Modeling Foxg1 regulation of L1-mRNA" section (from page 9, row 18, to page 10, row 28), trying to accompany the reader through complex correlative evidence which points to selective L1-mRNA downregulation in neuronogenic

© 2023. Published by The Company of Biologists under the terms of the Creative Commons Attribution License (https://creativecommons.org/licenses/by/4.0/).

progenitors. Finally, we reorganized the last part of the DISCUSSION (from page 18, row 18, to end), trying to simplify its logical architecture and make it more readable.

Responses to Reviewer 3

Reviewer 3 Advance Summary and Potential Significance to Field:
This study demonstrates the novel role of Foxg1 in regulating telencephalic development by exerting pleiotropic control over its progression. It explores the expression of L1 retrotransposons within the central nervous system (CNS) and suggests their potential contribution to genomic plasticity. By identifying putative Foxg1 binding motifs within L1 elements, the study proposes that Foxg1 may restrict the expression and activity of L1 retrotransposons in the telencephalon. Through in vivo and primary neural culture experiments using loss- and gain-of-function approaches, the study confirms this prediction, showing that Foxg1-dependent transcriptional repression of L1 occurs specifically in neopallial neuronogenic progenitors and post-mitotic neurons. Additionally, the study reveals a physical interaction between Foxg1 and L1-mRNA, with Foxg1 positively impacting neonatal neopallium L1-DNA content.
Surprisingly, Foxg1 antagonizes the retrotranscription-suppressing activity of Mov10 and Ddx39a helicases on L1 elements. This study establishes Foxg1 as the first CNS patterning gene with bimodal regulation of retrotransposons, both limiting and promoting L1 transcription and amplification within a specificdomain of the developing mouse brain.

We thank Reviewer 3 for her/his appreciation of our work.

Reviewer 3 Comments for the Author:
The authors have addressed most of my previous comments. However, they should revise the text to ensure their study is put in the context of the previously known literature of this context.

For example, this study significantly advances our understanding of neurodevelopmental processes by elucidating the intricate interplay between Foxg1 and L1 retrotransposons in the context of telencephalic development. The findings shed light on the regulatory mechanisms governing genomic plasticity within the central nervous system (CNS), a crucial aspect of neurodevelopmental processes. By identifying Foxg1 as a key regulator of L1 retrotransposon activity through both transcriptional repression and promotion, the study highlights the multifaceted role of this transcription factor in shaping the molecular landscape of the developing brain.

Moreover, the study's emphasis on the specific localization of Foxg1-dependent L1 repression to neopallial neuronogenic progenitors and post-mitotic neurons underscores the importance of spatial and temporal regulation in neurodevelopment. This insight contributes to our broader understanding of how gene regulatory networks orchestrate regional patterning and cellular differentiation within the developing CNS.

Furthermore, the unexpected discovery of Foxg1's interaction with L1-mRNA and its role in modulating L1-DNA content adds a new dimension to our understanding of retrotransposon regulation in neurodevelopment. By revealing Foxg1's ability to counteract the suppressive activities of Mov10 and Ddx39a helicases on L1 elements, the study highlights the complexity of the regulatory networks involved in retrotransposon biology.

How these aspects are related to previous findings of transcription factors, retrotransposons and cell- fate needs to be properly highlgihted.

While reorganizing the DISCUSSION, we paid special attention to concisely describe the "take home" messages originating from our study, and distinguish really novel findings (Foxg1 control over L1-mRNA and -DNA levels) from other findings which represent (1) a generalization to rodents of phenomena already described in humans (i.e. progressive L1-mRNA increase along the neuronogenic axis), or (2) a confirmation of similar results previously obtained in rodents (late-gestational increase of L1-DNA). We added one key reference (Garza et al., 2023), with single cell

© 2023. Published by The Company of Biologists under the terms of the Creative Commons Attribution License (https://creativecommons.org/licenses/by/4.0/).

L1-mRNA evaluation in human embryonic NSCs, NPs and neurons, we missed in previous, v2 version of the manuscript.

Concerning mechanistic aspects of Foxg1 control over L1 biology, we had already detailed functional similarities and differences between Foxg1 and other key transcription factors controlling L1 expression, such as Sox2. Moreover, as for Foxg1 impact on L1-DNA content, we had explicitly mentioned three established inhibitors of retrotransposition, through which Foxg1 can act. These are Apobec1 deaminase, whose mRNA is halved in Foxg1-OE neurons, and Mov10a and Ddx39a helicases, namely two components of Foxg1 interactome.

Regarding functional meaning of Foxg1 modulation of L1 elements, at the moment it is hard to make precise statements. In this respect, we had already intersected established Foxg1-LOF/OE phenotypes with neurodevelopmental abnormalities stemming from generalized L1 knock-down (as recently described by Mangoni et al, 2023), and, on the basis of that, we could anticipate a likely L1 role in mediating Foxg1 control over three key aspect of neopallial histogenesis.

We did our best to accurately contextualize our findings in previous literature on TFs of neurodevelopmental interest and L1 elements, within word count constrainsts granted us by the Journal. We do hope to have satisfied Reviewer 3's requests.

Third decision letter

MS ID#: DEVELOP/2023/202292

MS TITLE: Foxg1 bimodally tunes L1-mRNA and -DNA dynamics in the developing murine neocortex

AUTHORS: Gabriele Liuzzi, Osvaldo Artimagnella, Simone Frisari, and Antonello Mallamaci
ARTICLE TYPE: Research Article

I am delighted to tell you that your manuscript has been accepted for publication in Development, pending our standard ethics checks.

© 2023. Published by The Company of Biologists under the terms of the Creative Commons Attribution License (https://creativecommons.org/licenses/by/4.0/).
